# Total Synthesis and Biological Profiling of Putative (±)-Marinoaziridine B and (±)-*N*-Methyl Marinoaziridine A

**DOI:** 10.3390/md22070310

**Published:** 2024-07-03

**Authors:** Anđela Buljan, Višnja Stepanić, Ana Čikoš, Sanja Babić Brčić, Krunoslav Bojanić, Marin Roje

**Affiliations:** 1 Laboratory for Chiral Technologies, Scientific Center of Excellence for Marine Bioprospecting-BioProCro, Ruđer Bošković Institute, Bijenička cesta 54, 10000 Zagreb, Croatia; andjela.buljan@irb.hr; 2 Laboratory for Machine Learning and Knowledge Representation, Division of Electronics, Ruđer Bošković Institute, Bijenička cesta 54, 10000 Zagreb, Croatia; visnja.stepanic@irb.hr; 3 NMR Centre, Ruđer Bošković Institute, Bijenička cesta 54, 10000 Zagreb, Croatia; ana.cikos@irb.hr; 4 Laboratory for Aquaculture Biotechnology, Scientific Center of Excellence for Marine Bioprospecting-BioProCro, Ruđer Bošković Institute, Bijenička cesta 54, 10000 Zagreb, Croatia; sanja.babic@irb.hr (S.B.B.); krunoslav.bojanic@irb.hr (K.B.)

**Keywords:** marinoaziridines, total synthesis, antibacterial activity, antiproliferative activity, cytotoxicity, bioactivity profiling, ADMET, in silico activity, marine natural products

## Abstract

The total synthesis of two new marine natural products, (±)-marinoaziridine B **7** and (±)-*N*-methyl marinoaziridine A **8**, was accomplished. The (±)-marinoaziridine **7** was prepared in a six-step linear sequence with a 2% overall yield. The key steps in our strategy were the preparation of the chiral epoxide (±)-**5** using the Johnson Corey Chaykovsky reaction, followed by the ring-opening reaction and the Staudinger reaction. The *N*,*N*-dimethylation of compound (±)-**7** gives (±)-*N*-methyl marinoaziridine A **8**. The NMR spectra of synthetized (±)-marinoaziridine B **7** and isolated natural product did not match. The compounds are biologically characterized using relevant in silico, in vitro and in vivo methods. In silico ADMET and bioactivity profiling predicted toxic and neuromodulatory effects. In vitro screening by MTT assay on three cell lines (MCF-7, H-460, HEK293T) showed that both compounds exhibited moderate to strong antiproliferative and cytotoxic effects. Antimicrobial tests on bacterial cultures of *Escherichia coli* and *Staphylococcus aureus* demonstrated the dose-dependent inhibition of the growth of both bacteria. In vivo toxicological tests were performed on zebrafish *Danio rerio* and showed a significant reduction of zebrafish mortality due to *N*-methylation in (±)-**8**.

## 1. Introduction

A large number of natural products isolated from marine organisms often possess considerable bioactivity, such as antibiotic, antiviral, anticancer, neurodegenerative or anti-inflammatory properties, making them an invaluable source of pharmaceuticals and medicinal products, but their availability and usability have been limited [1,2,3,4,5,6,7]. Bioprospecting as a concept of sustainable use of biodiversity offers a way to solve these issues, while preserving biological resources. Screening natural resources for small molecules and macromolecules by collecting their biochemical and genetic information could ultimately lead to their development into commercially valuable products for pharmaceutical, food, cosmetic and other applications [8,9]. Natural products continue to be the main source of novel small molecule drugs, including their semi-synthetic derivatives and scaffolds inspired by them [10,11,12,13,14,15,16,17].

**Figure 1:**
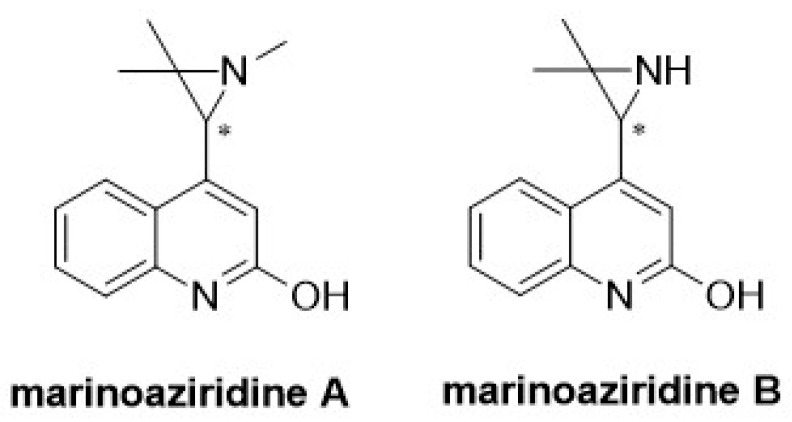
Chemical structures of marinoaziridines A and B as determined by Fenical et al. [18].

As a result of marine bioprospecting, Fenical and co-workers recently reported the structures of two new marine natural products, chiral alkaloids marinoaziridines A and B (Figure 1), which were isolated from Gram-negative bacteria of the order *Cytophagales* found in marine sediment [18].

The structural analysis of the isolated alkaloids was carried out using 1D and 2D NMR and MS techniques. The proposed structures of the natural products marinoaziridines A and B belong to a large family of quinolin-2(1*H*)-ones that have a specific cyclic amide functionality that can exist in two tautomeric forms, the lactim (enol) and lactam (keto) forms [19].

**Scheme 1:**
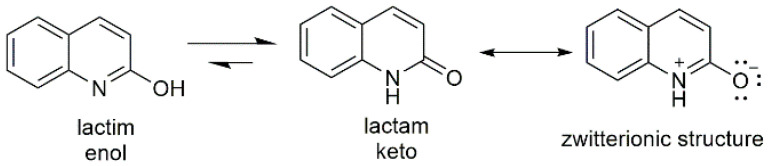
Keto-enol tautomerism of quinoline-2(1*H*)-one.

**Scheme 2:**
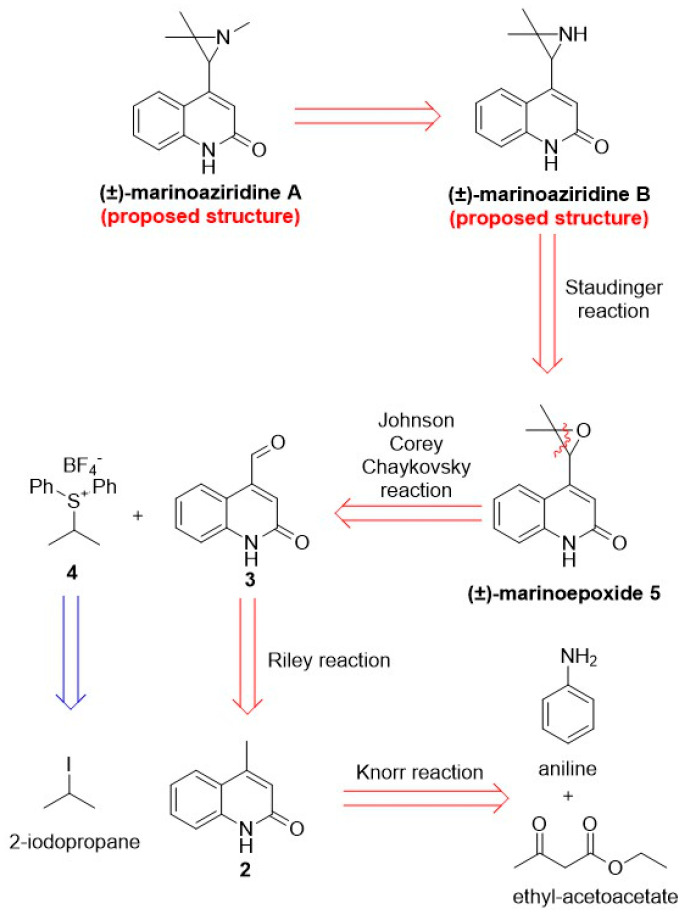
Retrosynthetic analysis of the proposed structures of (±)-marinoaziridines A and B.

Fenical and co-workers have described the structures of isolated marine natural products as enol forms (Figure 1) [18]. The literature suggests that the keto form is more stable than the enol form because the solvated lactam is stabilized by resonance with a zwitterionic structure (Scheme 1). Therefore, polar solvents should stabilize this zwitterionic resonance and decrease the energy of the lactam relative to the lactim tautomer [19,20,21]. Previously, we established a three-step protocol for synthesizing marinoepoxides as precursors in the total synthesis of marinoaziridines [22]. During this research, we verified the existence of a keto form in the quinolin-2(1*H*)-one intermediate through NMR analysis, which is consistent with literature. In light of this discovery, we have revised our approach to synthesize the final product in the keto form following Scheme 2.

The proposed marinoaziridines, aside from featuring the pharmacologically intriguing quinolin-2(1*H*)-one ring, also contain an aziridine ring commonly found in bioactive natural and synthetic molecules [23,24,25]. They possess one center of chirality, which allows for the existence of two stereoisomers (enantiomers) for each product, possibly associated with different and selective biological activity. The absolute configuration of the isolated products was determined by comparing their optical rotation (OR) with that of phenyl aziridine enantiomer, although the absolute OR values found are very different: [α]_D_^21^—6 for both natural products, and [α]_D_^21^—45.5 for (*R*)-2-phenyl aziridine. It is also surprising that the circular dichroism (CD) spectrum recorded for the two natural products was negligible. Based on the results of limited antibacterial tests on *Pontibacillus* sp. and *Vibrio shiloi*, the authors reported that the isolated products had no antibiotic activity [18]. 

Since the new marinoaziridines have unusual structural features and are only found in nature to a limited extent, we have set ourselves a goal to develop protocols for total synthesis in order to produce these molecules in sufficient quantities for initial biological evaluation.

## 2. Results and Discussion 

### 2.1. Total Synthesis of (±)-Marinoaziridine B and (±)-N-Methyl Marinoaziridine A

As part of our ongoing research into the synthesis of marine natural products and their derivatives, we began with the total synthesis of (±)-marinoaziridines A and B. The retrosynthetic analysis of (±)-marinoaziridines A and B is shown in Scheme 2.

One of the key steps in the total synthesis of (±)-marinoaziridine B is the Staudinger reaction, which involves the (±)-marinoepoxide **5**. Intermediate (±)-**5** can be prepared by a convergent strategy of combining quinolin-2(1*H*)-on and isopropyl fragments via a Johnson Corey Chaykovsky epoxidation following the synthetic protocol described in our recently published article [22]. 

**Scheme 3:**
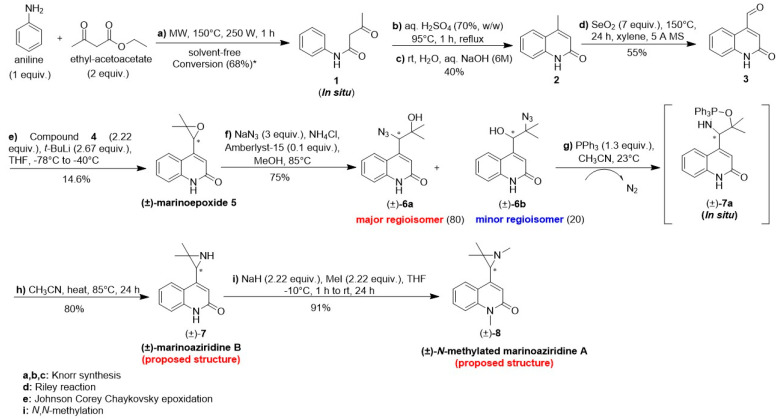
Total synthesis of (±)-marinoaziridine B **7** and (±)-*N*-methyl marinoaziridine A **8**.

**Figure 2:**
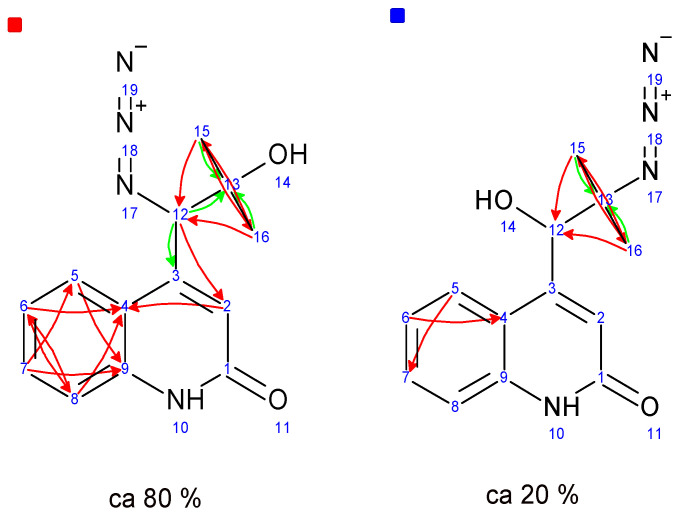
Structures, numbering and key ^1^H–^13^C HMBC contacts for the major (±)-**6a** (red) and minor (±)-**6b** (blue) regioisomer of (±)-**6**, arrows represent interactions through two (green) and three (red) bonds.

To date, efforts to synthesize (±)-marinoaziridine A have been unsuccessful, leading only to the preparation of its (±)-*N*-methyl derivative **8** (Scheme 3).

(±)-Marinoaziridine B **7** and (±)-*N*-methyl marinoaziridine A **8** were synthesized as shown in Scheme 3. Our synthetic endeavor began with the preparation of quinolin-2(1*H*)-one **2**. Aniline was first reacted with ethyl acetoacetate under microwave irradiation to give the corresponding compound **1**, which was then treated with aq. H_2_SO_4_ (70%, *w*/*w*) and converted by Knorr reaction to the lactam **2** with 40% yield and complete regioselectivity. The Knorr’s product **2** was then oxidized to the corresponding aldehyde **3** using the Riley oxidation [26] in 55% yield. Subsequently, the achiral sulfonium salt **4** was prepared from the commercially available 2-iodopropane and dimethyl sulfide in the presence of the AgBF_4_ as promoter in a 50% yield (Scheme S2) [22,27]. The reaction between the sulfonium salt **4**, the base-generated in situ sulfur ylide **4**′ and a carbonyl compound **3** according to the Johnson Corey Chaykovsky reaction gave the (±)-marinoepoxide **5** in a 14.6% yield [22]. The low yield is attributed to the poor solubility of the starting material, which stems from the presence of polar amide bonds. Additionally, performing the reaction at low temperatures exacerbates the issue. However, these low temperatures are necessary because the resulting ylide is unstable above −40 °C. The (±)-marinoepoxide **5** was then treated with NaN_3_ and NH_3_Cl in the presence of Amberlyst^®^ (Merck KGaA, Darmstadt, Germany) as an acid catalyst to give compound (±)-**6** in a good yield of 75%. Analysis of the 1D and 2D NMR spectra of (±)-**6** revealed two sets of resonance lines corresponding to the two regioisomers in a ratio of approximately 80:20 (Figure 2).

The major isomer (±)-**6a** (ca 80%) is characterized by the carbon chemical shifts of C-12 (68.6 ppm) and C-13 (73.6 ppm), which indicate the position of the hydroxy group at carbon C-13 (Figures S4 and S5). Similarly, the minor isomer (±)-**6b** (ca 20%) is characterized by carbon chemical shifts of C-12 (74.6 ppm) and C-13 (65.0 ppm), indicating the position of the hydroxy group at carbon C-12. Some of the carbon atoms, such as C-1 in the major regioisomer and C-1, C-3 and C-9 in the minor regioisomer, could not be assigned. 

Given that secondary azido alcohol (±)-**6b** requires activation before conversion to aziridine, we decided to optimize this step. We investigated a series of reagents (Table 1) to determine the optimal conditions for the efficient synthesis of compound (±)-**6a**. Generally, the ring opening of epoxides with azides proceeds via *S*_N_2 mechanism under neutral or basic conditions. Under acidic conditions, the epoxide’s oxygen is protonated, leading to a higher positive charge on the epoxide carbon with more substituents, thereby enhancing the azide attack. However, in the presence of aromatic ring as a substituent, electronic effects operate to route the incoming azide ion more to benzylic position due the stabilization of positive charge by resonance [28,29,30,31]. Using NaN_3_ in the presence of NH_4_Cl as Lewis acid and Amberlyst^®^ as acid catalyst in methanol afforded very good regioselectivity (80:20) for the product (±)-**6a** in good yield (60%), making these the best conditions tested (entry iv). NH_4_Cl coordinates to the epoxide [32] and thereby favors the regioselective attack at the α-position of the (±)-marinoepoxide **5**. Furthermore, upon the addition of 18-crown-6-ethers as a catalyst, the regioselectivity increased a slight to compound (±)-**6b**. The conversions did not improve using MeOH/H_2_O, 1/1 (entry ii. and iii.), and the regioselectivity is modest. The alterations in regioselectivity can be attributed to a slight polarity change at the interface of aqueous and organic phases [32]. Et_2_AlN_3_ (entry v) was determined to be ineffective in the desired transformation.

The mixture of azidoalcohols (±)-**6a**/**6b** was further reduced in the presence of PPh_3_ to give the intermediate (±)-**7a**, which was finally converted to (±)-marinoaziridine B **7**, by the thermal cyclization process. Under the described reaction conditions, no conversion of the regioisomer (±)-**6b** into aziridine product was observed. The proposed structure of (±)-marinoaziridine B **7** contains two nitrogen atoms (N1 in the quinoline ring, N2 in the aziridine ring), and both of them can be protonated; the selective methylation of the aziridine nitrogen atom is required to prepare marinoaziridine A. Unfortunately, the restricted selection of solvents and reagents led to the failure of the selective methylation. Aziridines are generally reactive compounds that, in acidic and basic environments, prefer to open a ring with a nucleophile. To prevent the aziridine ring from opening in this situation, methylation reactions must be ensured under mild conditions. Second, as was already indicated, quinolines’ solubility is extremely low in the solvent used in these processes. The aziridine nitrogen atom was successfully methylated when a methyl group was added to the amide bond, increasing its solubility. (±)-marinoaziridine B **7** was converted to (±)-*N*-methyl marinoaziridine A **8**, in the presence of NaH as base and MeI as reagent in 91% yield. 

The ^1^H and ^13^C NMR spectra of our synthetic (±)-marinoaziridine B **7** were not identical to the reported spectra of the authentic isolated marinoaziridine B. A closer look at the structure of marinoaziridine B (Figure 1) immediately reveals that it can exist in different tautomeric forms, which could explain this difference. As already mentioned, natural marinoaziridine B was isolated from marine sediment Gram-negative bacteria and described as enol form [18], whereas our NMR data for synthesized (±)-marinoaziridine B in CDCl_3_ at 25 °C correspond exclusively to the keto form. 

Because of the discovered discrepancy, we performed a full NMR structure elucidation of our synthetic (±)-marinoaziridine B **7** using 2D NMR to extract the connectivities, unambiguously confirm the structure and correctly assign all resonance lines. The results showed beyond doubt that the structure of our synthetic (±)-marinoaziridine B is as shown in Scheme 2. Having established that our synthetic sample has the structure of reported marinoaziridine B, we started looking more closely into structural errors in the natural sample NMR analysis. A comparison of all extracted chemical shifts with the previously reported data presented in Tables S1 and S2 shows significant differences, most notably in the proton chemical shift of H-12 and the carbon chemical shifts of C-12 and C-14. 

A statistical analysis of the experimental ^1^H and ^13^C chemical shifts values reported for Fenical’s marinoaziridine B and synthesized (±)-marinoaziridine B **7** (Figures S16 and S17) reveals a very low linear correlation (R^2^ = 0.965 for ^1^H and R^2^ = 0.9677 for ^13^C). The ^1^H and ^13^C NMR chemical shifts in the aromatic region of synthetic (±)-**7** were somewhat consistent with those reported for natural marinoaziridine B. However, there were large discrepancies in the chemical shifts in the aziridine ring region. The largest deviation between the two sets was found for the hydrogen atom at the chiral C-12 centre, which exhibited a chemical shift of 4.27 ppm in Fenical’s marinoaziridine B versus 3.25 ppm in the experimental spectra for synthetic (±)-marinoaziridine B **7**. 

The discrepancy in NMR spectra between the synthetic material and the natural product points toward the structural errors in the proposed structure of the natural product and, therefore, requires a revision of the proposed structure. Further investigations in this direction are currently underway and will be reported on in due course.

### 2.2. Biological Evaluation of Newly Synthesized Compounds: (±)-**7** and (±)-**8**

#### 2.2.1. In Silico ADMET and Bioactivity Profiling

**Figure 3:**
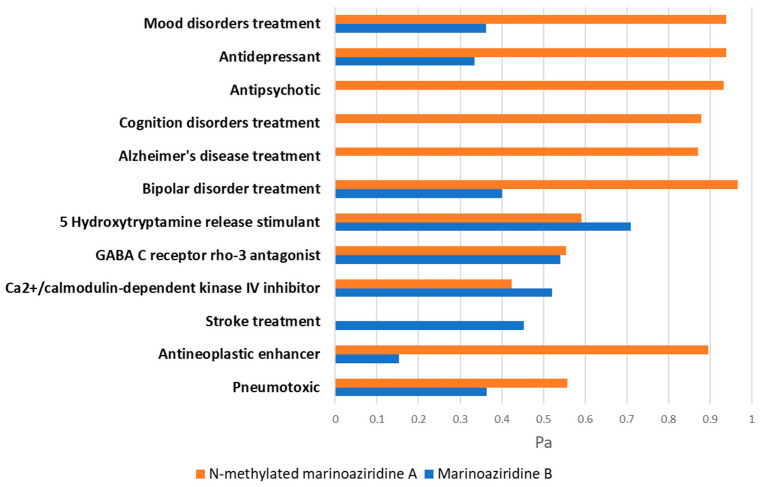
The biological activities of synthesized products (±)-**7** and (±)-**8** predicted by the program PASS with Pa ≥ 0.5.

Compounds (±)-**7** and (±)-**8** are drug-like molecules with a good estimated pharmacokinetic profile. They comply with Lipinski’s and Veber’s rules. Both molecules (±)-**7** and (±)-**8** are predicted to be moderately lipophilic (logP~1.6), water-soluble and membrane-permeable molecules (Table S3). The pK_a_ value for compounds (±)-**7** and (±)-**8** is estimated by the ADMET Predictor/ChemAxon modules to be 8.24/8.43 and 6.60/5.70, respectively. These pK_a_ predictions indicate that the proposed structures, although having a basic aziridine nitrogen, are expected to be largely neutral at a physiological pH of 7.4, allowing them to readily pass through membranes. Both proposed structures are expected to bind to blood plasma proteins to a small extent (the feature hum_fup%, Table S3) and to be transported mainly by entry into blood cells (RBP and Bioconcn). They are predicted to cross the blood–brain barrier (BBB) and remain in the brain, supporting the likelihood that they may have neuromodulatory effects predicted by the PASS 2020 program (Table S3, Figure 3).

In terms of adverse toxic effects, based on the ADMET Predictor results, the synthesized products could exert some mutagenic effects, and compound (±)-**7** could have adverse developmental effects, while its derivative (±)-**8** is likely to be an hERG inhibitor (Table S4). According to the PASS program [33], these molecules may exhibit respiratory toxicity.

The PASS program and the SwissTargetPredictor web server were used for in silico biological screening [33,34]. The structural comparison with 327,719 already known active compounds on 2092 human proteins by SwissTargetPrediction revealed kinases as potential targets for both marinoaziridines, although the structural likeness with known kinase inhibitors is low. According to PASS, neuromodulatory effects are predicted for both alkaloids (Figure 3). For derivative (±)-**8**, PASS predicted six pharmacological effects on neurodegenerative and neuropsychiatric disorders with a probability of being active Pa ≥ 0.5. In addition, PASS predicted three mechanisms of action for this derivative relevant to neuromodulatory effect: serotonin (5-hydroxytryptamine) release stimulant effect and gamma-aminobutyric acid receptor subunit rho-3 (GABRR3, Uniprot: A8MPY1) ion channel antagonist as well as the inhibitor of Ca^2+^/calmodulin-dependent kinase IV (CAMK4, Uniprot: Q16566) [35,36].

The predicted toxic and neuromodulatory effects may be relevant to the observed embryotoxicity in zebrafish (Table S6). In addition to the effect on the central nervous system, PASS estimated a moderate cardiovascular effect for (±)-marinoaziridine B **7** and a substantial likelihood probability of an anticancer effect for its methylated derivative (±)-**8**.

#### 2.2.2. Antiproliferative and Cytotoxic Effect In Vitro

The racemic mixtures of the newly synthesized compounds (±)-**7** and (±)-**8** were tested in vitro for their antiproliferative activity in two human cancer cell lines MCF-7 (breast cancer) and H-460 (lung cancer), as well as in the human non-tumor cell line HEK2933T (embryonic kidney) (Table 2). The results of these assays are listed in Table S5 and show that both compounds exhibited moderate to strong antiproliferative and cytotoxic effects (Figure S20).

#### 2.2.3. Antibacterial Activity In Vitro

The antibacterial activity of compounds (±)-**7** and (±)-**8** was evaluated using *E. coli* (Gram-negative bacteria) and *S. aureus* (Gram-positive bacteria) in the concentration range of 0.1–100 µg/mL for compound (±)-**7** and 0.3–280 µg/mL for compound (±)-**8**. Both tested compounds showed a dose-dependent inhibition of the growth of both bacteria, but the minimum inhibitory concentration (MIC) was observed only with compound (±)-**7** with *S. aureus* (50 µg/mL).

#### 2.2.4. Evaluation of Embryotoxicity Using Zebrafish (*Danio rerio*) In Vivo Model

The embryotoxicity of compounds (±)-**7** and (±)-**8** was investigated using the zebrafish *D. rerio*. Zebrafish embryos were exposed to three concentrations (0.4, 0.2 and 0.1 mM) of both compounds up to 96 h post-fertilization. None of the exposed embryos survived exposure to compound (±)-**7**, even at the lowest tested concentration (0.1 mM). Conversely, its derivative (±)-**8** at a concentration of 0.4 mM caused 77 ± 6% of mortality and 100% of developmental abnormalities including scoliosis, pericardial oedema, yolk sac area haemorrhage and yolk sac oedema (Figure S21). Furthermore, none of the exposed specimens hatched. At lower tested concentrations (0.2 mM and 0.1 mM), compound (±)-**8** did not induce mortality, however, the hatching success rate significantly decreased to 23 ± 6% (Table S6). No mortality or developmental abnormalities were observed within the control group.

## 3. Materials and Methods

### 3.1. Synthesis

Unless otherwise stated, all reactions were carried out under an atmosphere of argon. All solvents were either puriss p.a. quality or distilled over appropriate drying reagents. Reactions were performed using glassware that was either flame-dried under vacuum or oven-dried at 120 °C for 12 h. Air- and moisture-sensitive liquids and solutions were transferred via syringe. All other reagents were purchased from commercial sources and used without purifications. For the chromatographic separation silica gel (Fluka Analytical, 35–75 μm) was used. Analytical thin layer chromatography was performed on silica gel plates (DC-Alufolien-Kieselgel F_254_, Merck KGaA, Darmstadt, Germany). Visualization was carried out under ultra-violet irradiation (254 nm).

Compounds **1**–**5** were synthesized according to a previously reported synthetic protocol [22].

### 3.2. Structure Characterization

All solvents used for NMR sample preparation were purchased from EurIsotop (Saint-Aubin, France). Full ^1^H and ^13^C assignments were made on the basis of one- and/or two-dimensional NMR spectra (^1^H, ^13^C, COSY, NOESY, HSQC and HMBC), which were recorded on Bruker Avance AV600 with a 5 mm diameter BBO probe equipped with a *z*-gradient accessory. Spectra were acquired at 25 °C using standard Bruker pulse sequences on samples prepared in CDCl_3_ with TMS as an internal standard. NOESY spectra were obtained with the mixing time of 500 ms. Multiplets are abbreviated as follows: br—*broad*; s—*singlet*; d—*doublet*; t—*triplet*; q—*quartet*; m—*multiplet*. All NMR spectra with experimental parameters are included in Supporting Information.

The melting point for compound (±)-**8** was determined using an Electrothermal 9100 apparatus in open capillaries and is uncorrected.

High resolution mass spectrometry (HRMS) was performed on an Agilent 6545 LC/Q-TOF MS (Agilent Technologies, Santa Clara, CA, USA), operating in a positive electrospray ionization (ESI) mode. The samples were dissolved in a mixture of methanol/acetonitrile (9/1) and injected directly into the MS detector in 70% acetonitrile with 0.1% formic acid and 30% water with 0.1% formic acid, flow rate 0.2 mL min^−1^. Recording and data processing were performed using the Agilent MassHunter Acquisition Software (V.10.1), and Agilent MassHunter Qualitative Software (V.10.0).

### 3.3. Biological Activity

#### 3.3.1. In Silico Physicochemical and Bioactivity Profiling

The program ADMET Predictor version 10.0.0.11 (Simulation Plus Inc., Lancaster, CA, USA) [36] and ChemAxon Calculator Plugins [37] were used for the estimation of various molecular properties associated with drug-likeness and ADMET (Absorption, Distribution, Metabolism, Excretion and Toxicity) features of compounds. The ADMET Predictor built-in models were used to estimate water solubility (Sw), lipophilicity coefficient (logP), membrane (i.e., human effective jejunal) permeability (Peff), probability of passing blood-brain barrier (BBB), fraction of substance not bound to plasma proteins and free in plasma (%hum_fup) and ratio of drug concentration in blood and plasma (RBP). The ChemAxon Calculator Plugin was applied for the estimation of the acidity constants [38].

The biological profiles for target marinoaziridines were screened in silico by using the machine learning-based software PASS 2020 (Prediction of Activity Spectra for Substances) [33] and structural similarity founded web server SwissTargetPrediction [34].

The biological screening by PASS includes 1,945 recommended models for various pharmacological effects, mechanisms of action, toxic and adverse effects, antitargets, metabolism-related effects, gene expression regulation and transporter-related effects. The biological profile is given as a list of activities along with the probabilities of being active Pa and inactive Pi and with two conditions Pa > Pi and Pa ≥ 0.5. The SwissTargetPrediction web server predicts likely protein targets from the structural similarity of studied molecules to already known active compounds.

The SMILES strings of the compounds served as input for all computational programs used.

#### 3.3.2. In Vitro Testing

##### Antiproliferative Activity

Two racemic mixtures (±)-**7** and (±)-**8** were tested for their antiproliferative activity, with details on mass and concentration provided in Table 2.

**Table 2:** Tested Compounds.

Compound I.D.	Submitted MW ^a^	Stock Solution/Solvent
(±)-7	214.27	4 × 10^−2^ M/DMSO
(±)-8	242.32	4 × 10^−2^ M/DMSO

^a^ M.W. Molecular weight.

##### Cell Lines

The experiments were carried out on three human cell lines: H460 (lung cancer cell line), MCF-7 (breast cancer cell line) and HEK293T (embryonic kidney cell line).

##### Cell Culturing

Cells were cultured as monolayers and maintained in Dulbecco’s modified Eagle medium (DMEM), supplemented with 10% fetal bovine serum (FBS), 2 mM L-glutamine, 100 U/mL penicillin and 100 μg/mL streptomycin in a humidified atmosphere with 5% CO_2_ at 37 °C.

##### Proliferation Assays

Panel cell lines were inoculated onto a series of standard 96-well microtiter plates on day 0, at 1.5 × 10^4^ cells/mL or 2 × 10^4^ cells/mL (MCF-7), depending on the doubling times of the specific cell line. Test agents were then added in five 10-fold dilutions (10^−8^ to 10^−4^ M) and incubated for a further 72 h. The working dilutions were freshly prepared on the day of the test. The maximum concentration of the solvent for the test compounds never exceeded 0.5%. After 72 h of incubation the cell growth rate was evaluated by performing the MTT assay, which detects dehydrogenase activity in viable cells. The MTT Cell Proliferation Assay is a colorimetric assay system, which measures the reduction of a tetrazolium component (MTT) into an insoluble formazan product by the mitochondria of viable cells. For this purpose, the substance-treated medium was discarded, and 40 μL of MTT reagent was added to each well at a concentration of 0.5 µg/µL. After four hours of incubation the precipitates were dissolved in 160 μL of DMSO. The absorbance (A) was measured on a microplate reader at 570 nm. The absorbance is directly proportional to the cell viability. The percentage of growth (PG) of the cell lines was calculated according to one or the other of the following two expressions:

If (A_test_ − A_tzero_) ≥ 0 then: PG = 100 × (A_test_ − A_tzero_)/(A_cont_ − A_tzero_)

If (A_test_ − A_tzero_) < 0 then: PG = 100 × (A_test_ − A_tzero_)/A_tzero_

where:

A_tzero_ = the average absorbance before exposure of cells to the test compound;

A_test_ = the average absorbance after the desired period of time (72 h);

A_cont_ = the average absorbance after 72 h with no exposure of cells to the test compound.

Each test point was performed in quadruplicate in three individual experiments. The results are expressed as concentration–response graphs (Figure S20). A negative percentage indicates cytotoxicity following drug treatment where −100% shows no cells survived the treatment at the specific drug concentration. The results are also expressed as GI_50_, a concentration necessary for 50% of inhibition (Table S5). The GI_50_ values for each compound were calculated from dose response curves using linear regression analysis by fitting the test concentrations that give PG values above and below the respective reference value (e.g., 50 for GI_50_). Therefore, a “real” value for any of the response parameters was obtained only if at least one of the tested drug concentrations falls above, and likewise if at least one falls below the respective reference value. If, however, for a given cell line all of the tested concentrations produce PGs exceeding the respective reference level of effect, then the lowest tested concentration is assigned as the default value. In the screening data report, that default value is preceded by a “<“ sign, signifying that the “real” value is something “less-than” the lowest tested concentration. Likewise, if none of the tested concentrations produces the required PG reference level of effect or greater, then a “>“ sign precedes the printed default value (which is the highest tested concentration), signifying that the “real” value is something “greater-than” the highest tested concentration.

##### Antibacterial Activity

The antimicrobial activity of selected newly synthesized compounds was tested by broth microdilution method using two bacterial species, *Escherichia coli* NCTC 12241 and *Staphylococcus aureus* ATCC 6538, according to the CLSI guidelines [39]. Briefly, bacterial inocula were prepared from fresh overnight growth on TSA agar (Biolife, Milan, Italy) by turbidity adjustment (bioMérieux, Craponne, France) to a concentration of ~5 × 10^5^ CFU mL^−1^. Mueller-Hinton broth (Merck, Darmstadt, Germany) was used for testing antimicrobial activity with all assays employing positive (inoculated media without the tested compounds) and negative (sterile media only) controls and an external quality control using amoxicillin (Sigma–Aldrich, Darmstadt, Germany). The maximal concentration of the DMSO solvent was 1% (*v*/*v*), and samples were tested using ten 2-fold dilutions in a 96-well microtitre plate. All tests were performed in duplicates at 35 °C aerobically with results interpreted both visually and spectrophotometrically at 600 nm (OD 600) using a Tecan Infinite Pro microplate reader (Tecan, Grödig, Austria). The minimum inhibitory concentration (MIC) was recorded as the lowest concentration of sample that completely inhibited bacterial growth. Inoculum concentrations and purity were checked by isolation from wells and CFU counting.

#### 3.3.3. In Vivo Testing

##### Embryotoxicity Testing

Embryotoxicity testing was performed according to the standardized OECD 236, 2013 [40] using zebrafish *D. rerio* (wild WIK strain supplied by the European Resource Center, Karlsruhe Institute of Technology (KIT), Karlsruhe, Germany). Animal maintenance and spawning were performed in aquaria units approved by the Croatian Ministry of Agriculture (HR-POK-023) under conditions described in detail in our previous study [41]. The tested compounds (±)-**7** and (±)-**8** were dissolved in DMSO solvent (p.a.), after which they were diluted to the desired concentration using the artificial water. The concentration of DMSO in tested dilutions did not exceed 0.1%. Immediately upon spawning, embryos were exposed to 1 mL of the tested concentration. The negative control group was exposed to artificial water, while as solvent control, 30 embryos were exposed to 0.1% DMSO. Ten embryos were used in three replicates amounting to 30 embryos per tested concentration. Plates with embryos were incubated at 27.5 ± 0.5 °C (Phoenix Instrument incubator TIN-IN35, Naperville, IL, USA). After 96 h after exposure, larvae were inspected using an Olympus CKX41 (Olympus, Tokyo, Japan) inverted microscope with an associated Leica EC3 digital camera (Leica, Wetzlar, Germany) and LAS EZ 3.2.0 software for image analysis, when survival, incidence of developmental abnormalities, and hatching success were recorded.

## 4. Conclusions

To summarize, the first total synthesis of the natural product described in the literature as marinoaziridine B (±)-**7** was achieved in six overall steps from readily available aniline and ethyl acetoacetate in racemic form with an overall yield of 2%. The *N*,*N*-dimethylation of compound (±)-**7** gave its derivative, (±)-*N*-methyl marinoaziridine **8,** in 91% yield. The structure of the synthesized compound (±)-**7** was unequivocally confirmed to be identical to the previously reported structure of the isolated natural product. However, a comparative study of the published NMR chemical shifts of the isolated natural product with the NMR chemical shifts of our synthetic product (±)-**7** showed large differences that cannot be explained by different tautomeric forms and need to be further investigated. These investigations are already underway, and the results will be reported in due course. The preliminary in silico, in vitro and in vivo biological tests presented here were performed on racemic mixtures of compounds (±)-**7** and (±)-**8**. Because of their predicted potential for neuromodulatory effects, as well as their demonstrated antimicrobial activity along with the significant reduction of zebrafish mortality due to methylation, the next steps for compounds (±)-**7** and (±)-**8** would be to perform the biological tests on enantiomerically pure compounds followed by lead optimization through chemical derivatization.

## Figures and Tables

**Table 1:** Optimization of the (±)-marinoepoxide **5** ring-opening reaction.

Entry	Conditions	Yield ((±)-6a + (±)-6b)%	Ratio ((±)-6a/(±)-6b) ^a^
i.	NaN_3_ (2 equiv.), NH_4_Cl (2 equiv.), 18-crown-6 (0.05 equiv.), MeOH, 65 °C, 72 h	80	40/60
ii.	NaN_3_ (2 equiv.), NH_4_Cl (2 equiv.), 18-crown-6 (0.05 equiv.), MeOH/H_2_O (1/1), 65 °C, 72 h	77	42/58
iii.	NaN_3_ (2 equiv.), NH_4_Cl (2 equiv.), Amberlyst^®^ (0.1 equiv.) MeOH/H_2_O (1/1), 65 °C, 72 h	50	52/48
iv.	NaN_3_ (2 equiv.), NH_4_Cl (2 equiv.), Amberlyst^®^ (0.1 equiv.), MeOH, 65 °C, 72 h	75	80/20
v.	Et_2_AlN_3_ (2 equiv.), toluene, −10 °C to RT, 24 h	/	/

^a^ ratio of regioisomers determined by NMR spectroscopy.

## Data Availability

The data underlying this study are available in the published article and its Supporting Information.

## Supplementary Materials

The following supporting information can be downloaded at: https://www.mdpi.com/article/10.3390/md22070310/s1, Supporting Information contains summary of total synthesis including the details on chemical reactions and experimental analytical data obtained for all compounds, fully assigned NMR spectra of all synthesized compounds, comparison between synthesized compounds and exiting literature data, details on biological activity and references.
