# Supplementary material for: Total Synthesis and Biological Profiling of Putative (±)-Marinoaziridine B and (±)-N-Methyl Marinoaziridine A
Source: Mar Drugs. 2024 Jul 3;22(7):310. doi: 10.3390/md22070310 (PMC11278217; doi:10.3390/md22070310)
Supplement: Supplementary file 1 [file marinedrugs-22-00310-s001.zip › marinedrugs-3008265 S1.pdf]

Supporting Information

Total Synthesis and Biological Profiling of Putative (±)-Marinoaziridine B and (±)-*N*-Methyl Marinoaziridine A

Anđela Buljan [1], Višnja Stepanić [2], Ana Čikoš [3], Sanja Babić [4], Krunoslav Bojanić [4] and Marin Roje [1,*]

[1] Laboratory for Chiral Technologies, Scientific Center of Excellence for Marine Bioprospecting-BioProCro, Ruđer Bošković Institute, Bijenička cesta 54, 10000 Zagreb, Croatia; Andjela.Buljan@irb.hr, Marin.Roje@irb.hr
[2] Laboratory for Machine Learning and Knowledge Representation; Division of Electronics, Ruđer Bošković Institute, Bijenička cesta 54, 10000 Zagreb, Croatia; Visnja.Stepanic@irb.hr
[3] NMR Centre, Ruđer Bošković Institute, Bijenička cesta 54, 10000 Zagreb, Croatia; Ana.Cikos@irb.hr
[4] Laboratory for Biotechnology in Aquaculture, Scientific Center of Excellence for Marine Bioprospecting-BioProCro Ruđer Bošković Institute, Bijenička cesta 54, 10000 Zagreb, Croatia; Sanja.Babic@irb.hr, Krunoslav.Bojanic@irb.hr
* Correspondence: Marin.Roje@irb.hr; Tel.: (+385 1 4571 283)

Table of Contents

1. Research Methodology S1
2. Summary of the Total Synthesis........S2
3. Experimental Section........S3
3.1. Experimental procedures........S3
3.2. NMR analysis of synthetic intermediates and final products........S5
3.3. Comparison of synthetic and isolated marinoaziridne B with literature data S20
4. Biological activity........S24
4.1. *In silico* ADMET and bioactivity profiling........S24
4.2. Antiproliferative effect........S25
4.3. Testing of embryotoxicity using the zebrafish model organism (*Danio rerio)*........S26
5. References........S27

1. Research Methodology

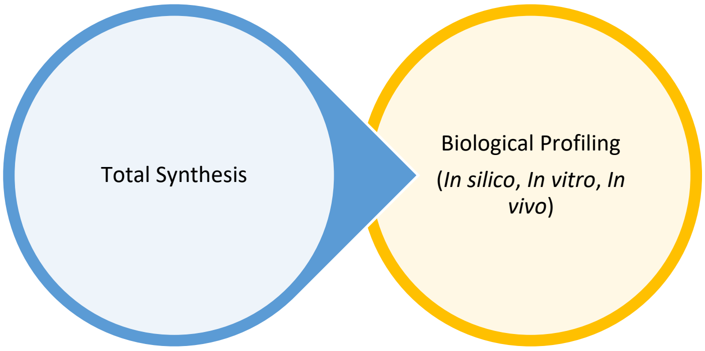

2. Summary of the total synthesis

The compounds **1-5** were synthesized to following our reported procedure [1].

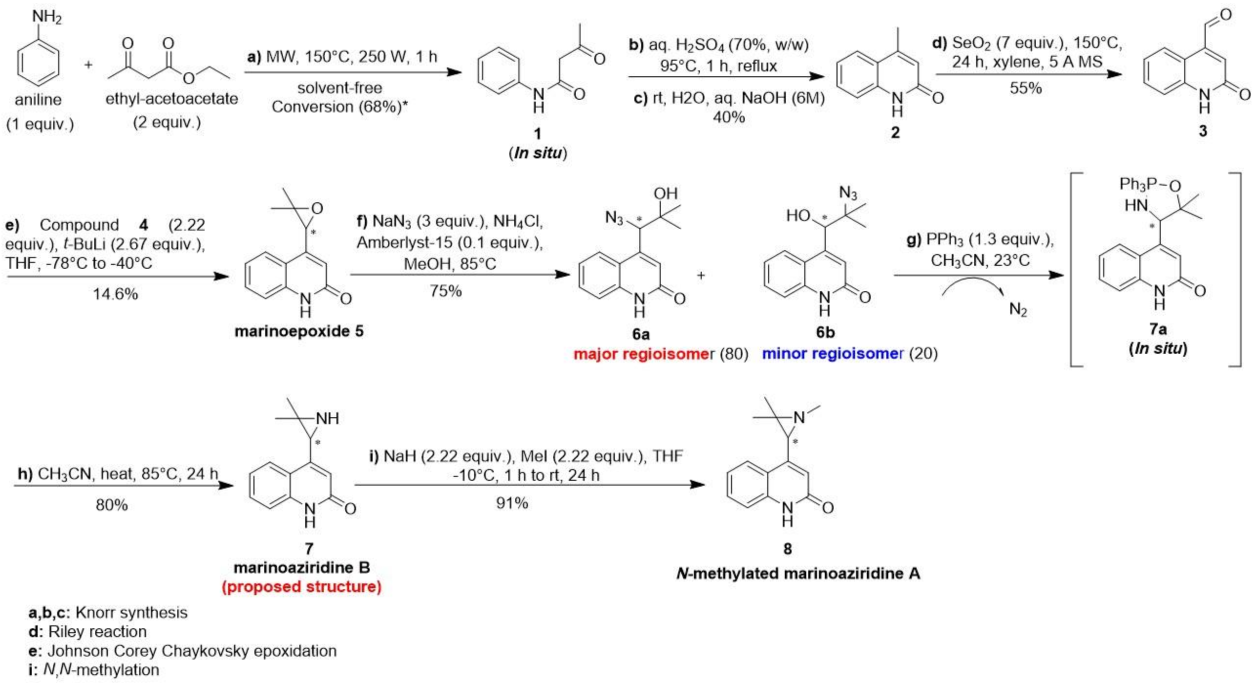

Scheme S1. Total synthesis of the proposed structure of marinoaziridine B (**7**) and *N*-methyl marinoaziridine A (**8**). Reagents and conditions: **a)** microwave-assisted synthesis, 150 °C, 1 h, keeping the power constant at 250 W, solvent-free, conversion of the starting material determined by HPLC analysis of the crude reaction mixture (68%), compound **1** was used in the next step without purification; **b)** aq. H_2SO_4 (70%, w/w, 2 equiv.), 95 °C, 1 h, **c)** then H_2O, aq. NaOH (6M), 23 °C, pH = 7, 40% (2 steps); **d)** SeO_2 (7 equiv.), xylene, 5 Å, powder, activated, 150 °C, 24 h, 55%; **e)** compound **4** (2.22 equiv.), *t*-BuLi (2.67 equiv.), THF, -78 °C to -50 °C, then H_20, aq. NH_4Cl, 14.6%; **f)** NaN_3 (3 equiv.), NH_4Cl (3 equiv.), Amberlyst®-15 (0.1 equiv.), MeOH, 85 °C, 24 h, 75% (**6a:6b**= 80:20, rr); **g)** PPh_3 (1.3 equiv.), CH_3CN, 23 °C; **h)** CH_3CN, heat, 85 °C, 24 h, 80%; **i)** i) NaH (2.22 equiv.), MeI (2.22 equiv.), THF, -10 °C, 1 h to rt, 24 h, 91%.

a. Synthesis of compound 4

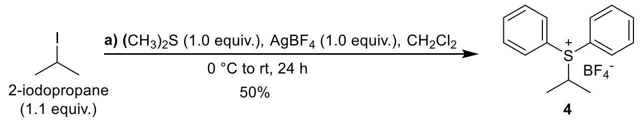

Scheme S2. Synthesis of compound **4**. Reagents and conditions: a) 2-iodopropane (1.1 equiv.), $(CH_3)_2S$ (1.0 equiv.), $AgBF_4$ (1.0 equiv.), CH_2Cl_2, 0 °C to rt, 24 h, 50% [1].

3. Experimental Section

3.1. Experimental procedures

The compounds **1-5** were synthesized to following our reported procedure [1].

❖Preparation of racemic quinolin-2(1*H*)-one azido alcohol 6a-b:

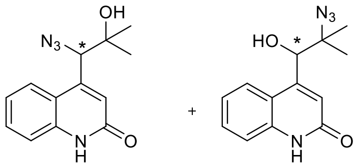

6a major regioisomer (80) **6b** minor regioisomer (20)

A closed tube was charged with compound **5** (0.160 g, 0.7 mmol), sodium azide (0.136 g, 2.1 mmol), ammonium chloride (0.112 g, 2.1 mmol) and Amberlyst®-15 (0.031 g, 0.1 equiv.) in methanol (3 mL) and was stirred at 85 °C until HPLC indicated complete consumption of starting materials. The mixture was then diluted with water. The aqueous layer was extracted with ethyl acetate (3 x 15 mL). The combined organic layers were washed with brine, dried over anhydrous Na_2SO_4, filtered and concentrated under reduced pressure. The regioisomeric side product was observed on the crude NMR (**6a**:**6b** = 80:20, *rr*). The crude product was purified *via* column chromatography (*flash* silica gel, dichloromethane/methanol = 25/1). Pure products **6a/6b** (0.144 g, 75%, 80:20, *rr*) were isolated as a light-yellow oils.

- **Data for 4-(1-azido-2-hydroxy-2-methylpropyl)quinolin-2(1*H*)-one 6a (major regioisomer):** light yellow oil; ^{1}H NMR ($CDCl_3$, 600 MHz) 11.23 – 11.10 (1H, m, H-10), 7.87 (1H, d, J_1 = 8.4 Hz, H-5), 7.55 (1H, ddd, J_1 = 8.8 Hz, J_2 = 7.0 Hz, J_3 = 1.2 Hz, H-7), 7.38 (1H, dd, J_1 = 7.7 Hz, J_2 = 1.1 Hz, H-8), 7.28 (1H, ddd, J_1 = 8.8 Hz, J_2 = 7.3 Hz, J_3 = 1.5 Hz, H-6), 6.94 (1H, s, H-2), 5.10 (1H, s, H-12), 1.93 (1H, s, H-14), 1.31 (3H, s, H-15), 1.30 (3H, s, H-16); ^{13}C ($CDCl_3$, 151 MHz) 138.08 (C, C-9), 130.99 (CH, C-7), 124.84 (CH, C-5), 122.77 (CH, C-6), 121.98 (CH, C-2), 119.25 (C, C-4), 116.71 (CH, C-8), 73.62 (C, C-13), 68.62 (CH, C-12), 27.03 (CH_3, C-15), 26.17 (CH_3, C-16); HRMS *m/z* 259.11968 (calculated for $C_{13}H_{14}N_4O_2$ $[M+H]^+$ 259.11950).

- **Data for 4-(2-azido-1-hydroxy-2-methylpropyl)quinolin-2(1*H*)-one 6b (minor regioisomer):** light yellow oil; ^{1}H NMR ($CDCl_3$, 600 MHz) 10.98 – 11.08 (1H, m, H-10), 7.83 (1H, d, J_1 = 8.1 Hz, H-4), 7.52 (1H, br t, J_1 = 8.1 Hz, H-7), 7.34 (1H, d, J_1 = 8.1 Hz, H-8), 7.25 (1H, t, J_1 = 7.3 Hz, H-6), 6.99 (1H, s, H-2), 5.19 (1H, d, J_1 = 2.9 Hz, H-12), 2.57 (1H, br d, J_1 = 3.7 Hz, H-14), 1.35 – 1.40 (6H, m, H-16, H-15); ^{13}C ($CDCl_3$, 75 MHz) 131.25 (CH, C-7), 125.07 (CH, C-5), 122.54 (CH, C-6), 121.04 (CH, C-2), 120.60 (C, C-4), 116.56 (CH, C-8), 74.64 (CH, C-12), 65.01 (C, C-13), 22.49 (CH_3, C-16), 22.33 (CH_3, C-15); HRMS *m/z* 259,11968 (calculated for $C_{13}H_{14}N_4O_2$ $[M+H]^+$ 259,11950).

❖Preparation of racemic 4-(3,3-dimethylaziridin-2-yl)quinolin-2(1*H*)-one, compound 7:

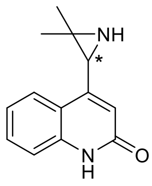

7
marinoaziridine B
(proposed structure)

A mixture of azido alcohols **6a/6b** (0.091 g, 0.33 mmol) and triphenylphosphine (0.102 g, 0.40 mmol) in dry acetonitrile (5 mL) was stirred at room temperature for 1h, and then the resulting mixture was stirred 24 h at 85°C, until TLC indicated complete consumption of starting materials. The mixture was then diluted with water. The aqueous layer was extracted with dichloromethane (3 x 15 mL). The combined organic layers were washed with brine, dried over anhydrous Na_2SO_4, filtered and concentrated under reduced pressure. The crude product was purified *via* column chromatography (*flash* silica gel, dichloromethane/methanol = 25/1). Pure product **7** (0.051 g, 80%) was isolated as yellow oil.

- **Data for racemic 4-(3,3-dimethylaziridin-2-yl)quinolin-2(1*H*)-one, compound 7:** yellow oil; ^{1}H NMR ($CDCl_3$, 600 MHz) 12.14 (NH, br s, H-10), 7.71 (1H, br d, J_1 = 7.7 Hz, H-5), 7.52 (1H, m, H-7), 7.50 (1H, m, H-8), 7.25 (1H, t, J_1 = 7.0 Hz, H-6),

6.81 (1H, s, H-2), 3.25 (1H, br s, H-12), 1.64 (3H, s, H-16), 0.98 (3H, s, H-15); ^{13}C ($CDCl_3$, 151 MHz) 164.16 (C, C-1), 149.03 (C, C-3), 138.32 (C, C-9), 130.63 (CH, C-7), 123.83 (CH, C-5), 122.70 (CH, C-6), 119.79 (C, C-4), 119.58 (CH, C-2), 116.74 (CH, C-8), 43.03 (CH, C-12), 39.67 (C, C-14), 26.75 (CH_3, C-16), 19.16 (CH_3, C-15); HRMS *m/z* 215.11857 (calculated for $C_{13}H_{14}N_2O$ $[M+H]^+$ 215.11844).

❖ **Preparation of racemic 1-methyl-4-(1,3,3-trimethylaziridin-2-yl)quinolin-2(1*H*)-one, compound 8:**

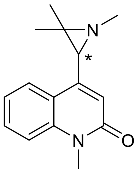

8
***N*-methylated marinoaziridine A**

To a flame dried Schlenk flask was added compound **7** (0.021 g, 0.09 mmol) which was stirred under vacuum for 0.5 h, and then suspended in dry THF (5 mL) under an atmosphere of argon. The mixture was cooled to -10 and then was added NaH (0.003 g, 0.20 mmol). The reaction mixture was stirred for 0.5 h before the addition of the methyl iodide (6 µL, 0.20 mmol). After the addition was complete, the solution was heated to room temperature until TLC indicated complete consumption of starting materials. The reaction was quenched by the addition of water (10 mL). The aqueous layer was extracted with dichloromethane (3 x 30 mL). The combined organic layers were washed with brine (20 mL), dried over anhydrous Na_2SO_4 and filtered. The solvent was removed in *vacuo* to give a yellow oil which was purified *via* column chromatography (*flash* silica gel, dichloromethane/methanol = 25/1). Pure product **8** (0.021, 91%) was isolated as a white solid.

- **Data for racemic racemic 1-methyl-4-(1,3,3-trimethylaziridin-2-yl)quinolin-2(1*H*)-one, compound 8:** white solid; mp 135–136 °C; ^{1}H NMR ($CDCl_3$, 600 MHz) 7.70 (1H, dd, J_1 = 7.9 Hz, J_2 = 1,3 Hz, H-5), 7.58 (1H, ddd, J_1 = 8.5 Hz, J_2 = 7.2 Hz, J_3 = 1.5 Hz, H-7), 7.39 (1H, d, J_1 = 8.4 Hz, H-8), 7.25 (1H, ddd, J_1 = 8.1 Hz, J_2 = 7.0 Hz, J_3 = 1.2 Hz, H-6), 6,85 (1H, d, J_1 = 1.1 Hz, H-2), 3,72 (3H, s, H-11), 2.61 (3H, s, -13H), 2.44 (1H, s, H-12), 1.51 (3H, s, H-15), 0.90 (3H, s, H-16); ^{13}C ($CDCl_3$, 151 MHz) 162.34 (C, C-1), 146.93 (C, C-3), 139.88 (C, C-9), 130.34 (CH, C-7), 124.56 (CH, C-5), 121.86 (CH, C-6), 120.76 (C, C-4), 119.91 (C, C-2), 114.53 (CH, C-8), 51.27 (CH, C-12), 43.09 (C, C-14), 39.47 (NCH_3, C-13), 29.25 (NCH_3, C11), 21.35 (CH_3, C-16), 17.34 (CH_3, C-15); HRMS *m/z* 243.14974 (calculated for $C_{15}H_{18}N_2O$ $[M+H]^+$ 243,14974).

3.2. NMR analysis of synthetic intermediates and final products

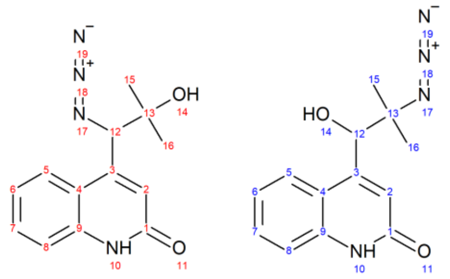

ca 80% ca 20%

Shift1 (ppm)	H's	Type	J (Hz)	Atom1	Multiplet1
1.298	3.000	s	-	16	M10
1.312	3.000	s	-	15	M09
1.373	6.000	m	-	16, 15	M19
1.929	1.000	s	-	14	M08
2.565	1.000	br d	3.7	14	M18
5.096	1.000	s	-	12	M07
5.192	1.000	d	2.9	12	M17
6.935	1.000	s	-	2	M06
6.991	1.000	s	-	2	M16
7.246	1.000	t	7.3	6	M15
7.277	1.000	ddd	8.8, 7.3, 1.5	6	M05
7.339	1.000	d	8.1	8	M14
7.376	1.000	dd	7.7, 1.1	8	M04
7.522	1.000	br t	8.1	7	M13
7.551	1.000	ddd	8.8, 7.0, 1.2	7	M03
7.834	1.000	d	8.1	5	M11
7.871	1.000	d	8.4	5	M02
11.033	1.000	m	-	10	M12
11.160	1.000	m	-	10	M01

Atom	Exp. Shift (ppm)	Multiplet	Component
16	1.30	M10	Major
15	1.31	M09	Major
14	1.93	M08	Major
12	5.10	M07	Major
2	6.94	M06	Major
6	7.28	M05	Major
8	7.38	M04	Major
7	7.55	M03	Major
5	7.87	M02	Major
10	11.16	M01	Major
15	1.37	M19	Minor
16	1.37	M19	Minor
14	2.57	M18	Minor
12	5.19	M17	Minor
2	6.99	M16	Minor
6	7.25	M15	Minor
8	7.34	M14	Minor
7	7.52	M13	Minor
5	7.83	M11	Minor
10	11.03	M12	Minor

Solvent	**CHLOROFORM-d**
Pulse Sequence	**zg30**
Temperature (degree C)	**25.000**
Acquisition Time (sec)	**1.3631**
Number of Transients	**32**
Points Count	**32768**
Sweep Width (Hz)	**12018.86**
Spectrum Offset (Hz)	**5390.2744**
Original Points Count	**16384**
Nucleus	**1H**
Frequency (MHz)	**600.1300**

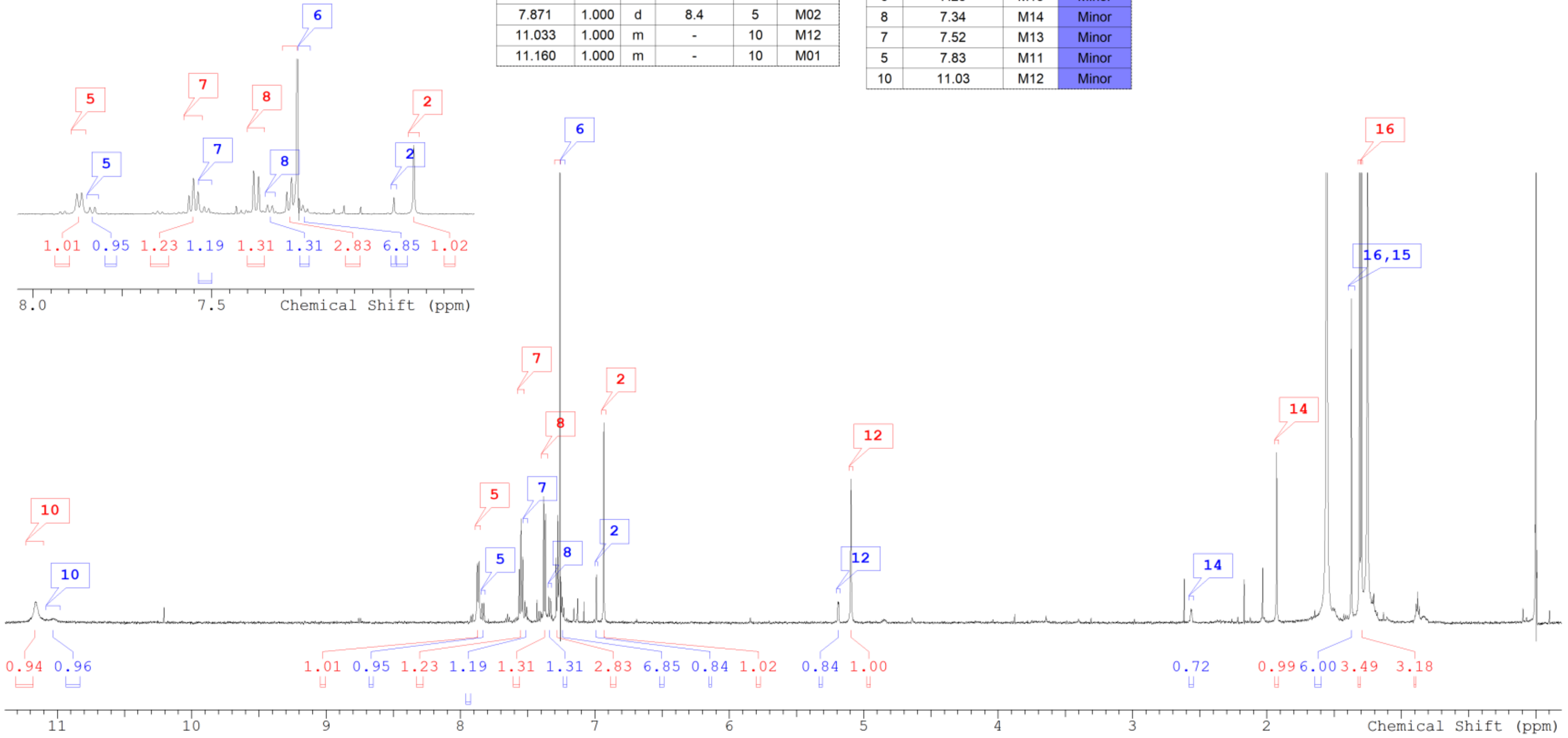

Figure S1. Structure, numbering and full assignment of ^{1}H NMR spectrum for **6a,b** in $CDCl_3$ at 25 °C.

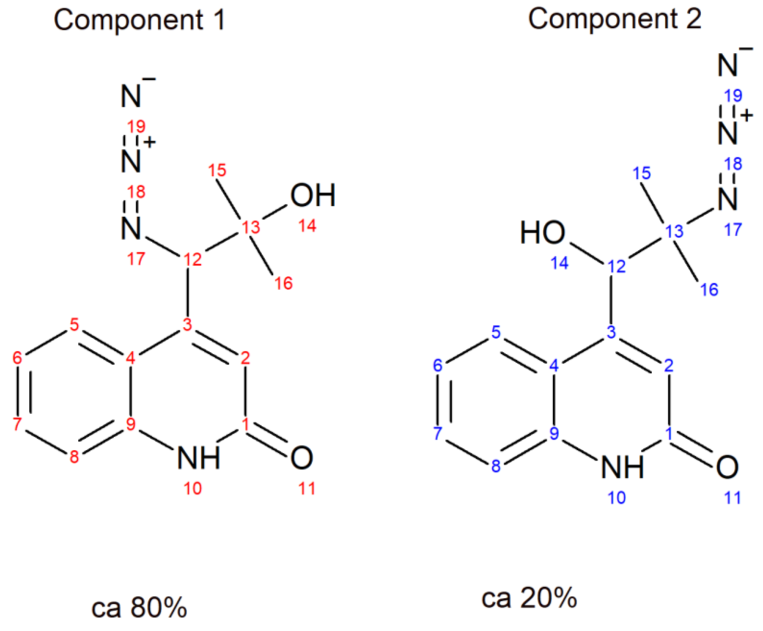

Atom	Exp. Shift (ppm)	Component
16	26.2	Component 1
15	27.0	Component 1
12	68.6	Component 1
13	73.6	Component 1
8	116.7	Component 1
4	119.2	Component 1
2	122.0	Component 1
6	122.8	Component 1
5	124.8	Component 1
7	130.6	Component 1
9	138.1	Component 1
1	-	Component 2
9	-	Component 2
3	-	Component 2
15	22.3	Component 2
16	23.4	Component 2
13	65.0	Component 2
12	74.1	Component 2
8	116.6	Component 2
4	120.6	Component 2
2	121.0	Component 2
6	122.5	Component 2
5	125.1	Component 2
7	131.3	Component 2

^{13}C NMR (151 MHz, CHLOROFORM-d): δ = 22.3 (15), 23.4 (16), 26.2 (16), 27.0 (15), 65.0 (13), 68.6 (12), 73.6 (13), 74.1 (12), 116.6 (8), 116.7 (8), 119.2 (4), 120.6 (4), 121.0 (2), 122.0 (2), 122.5 (6), 122.8 (6), 124.8 (5), 125.1 (5), 130.6 (7), 131.3 (7), 138.1 (9), 147.6 (3) ppm

Figure S2. Structure, numbering and full assignment of ^{13}C chemical shifts extracted from ^{1}H-^{13}C HSQCe and ^{1}H-^{13}C HMBC NMR spectra for **6a,b** in $CDCl_3$ at 25 °C.

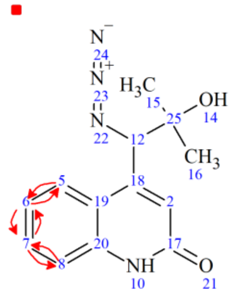

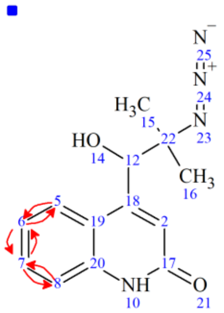

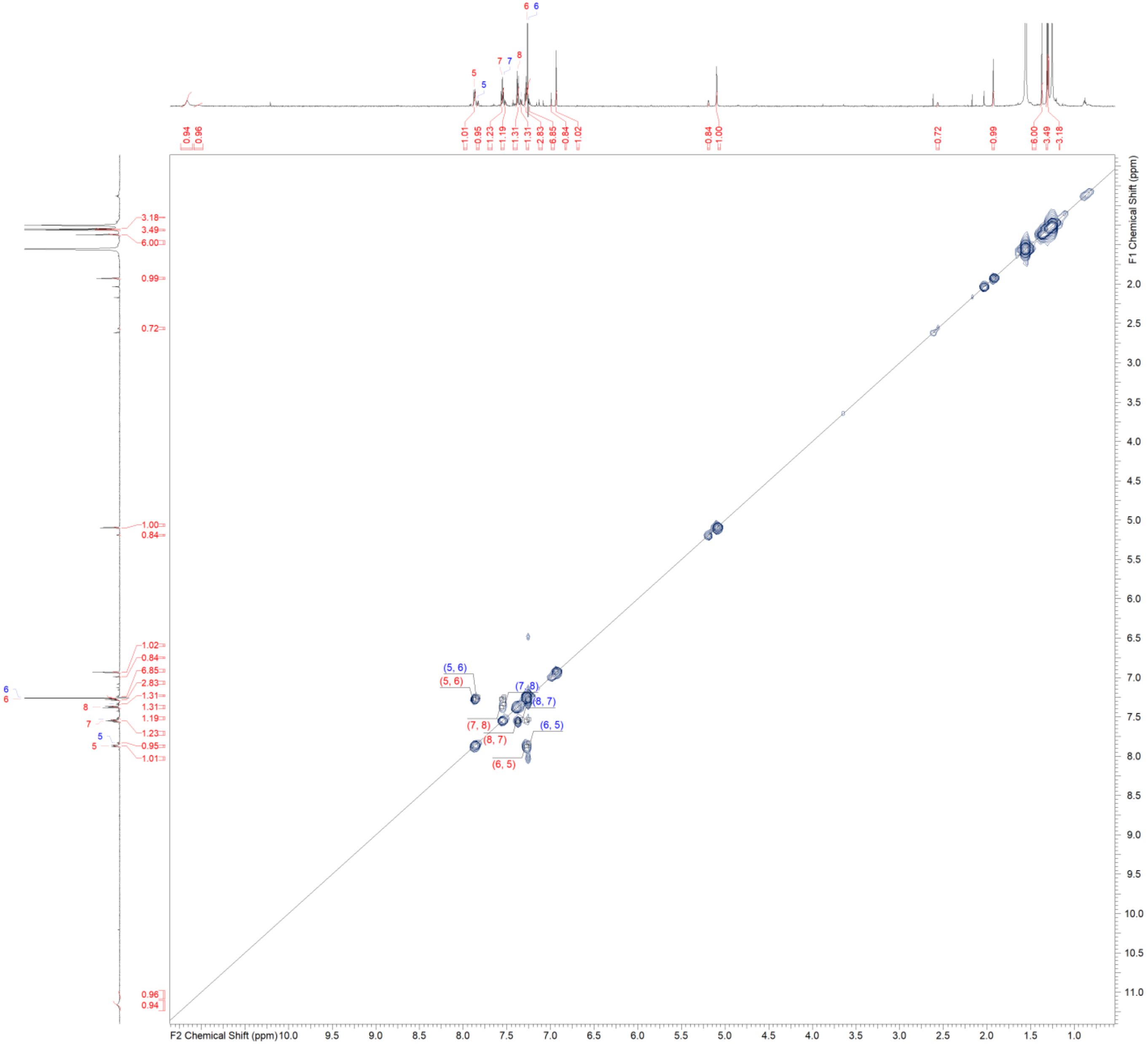

Acquisition Time (sec)	(0.1065, 0.0267)
Date	05 Apr 2022 21:13:18
Frequency (MHz)	(600.1300, 600.1300)
Nucleus	(1H, 1H)
Points Count	(2048, 2048)
Pulse Sequence	cosygpqf
Solvent	CHLOROFORM-d
Sweep Width (Hz)	(9610.69, 9597.45)
Number of Transients	4
Spectrum Type	COSY
Temperature (degree C)	25.000

Figure S3. Fully assigned ^{1}H-^{1}H COSY NMR spectrum of **6a,b** in $CDCl_3$ at 25 °C.

Component 1 ca 80%

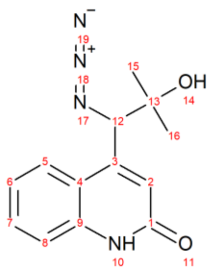

Component 2 ca 20%

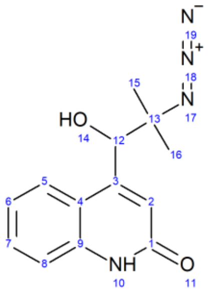

Acquisition Time (sec)	(0.1139, 0.0094)
Comment	MA 410 3 mg in 600 ul CDCl3
Frequency (MHz)	(600.1300, 150.9028)
Nucleus	(1H, 13C)
Origin	spect
Original Points Count	(1024, 256)
Points Count	(1024, 1024)
Pulse Sequence	hsqcedetgpsisp.2
Solvent	CHLOROFORM-d
Sweep Width (Hz)	(8984.02, 27138.29)

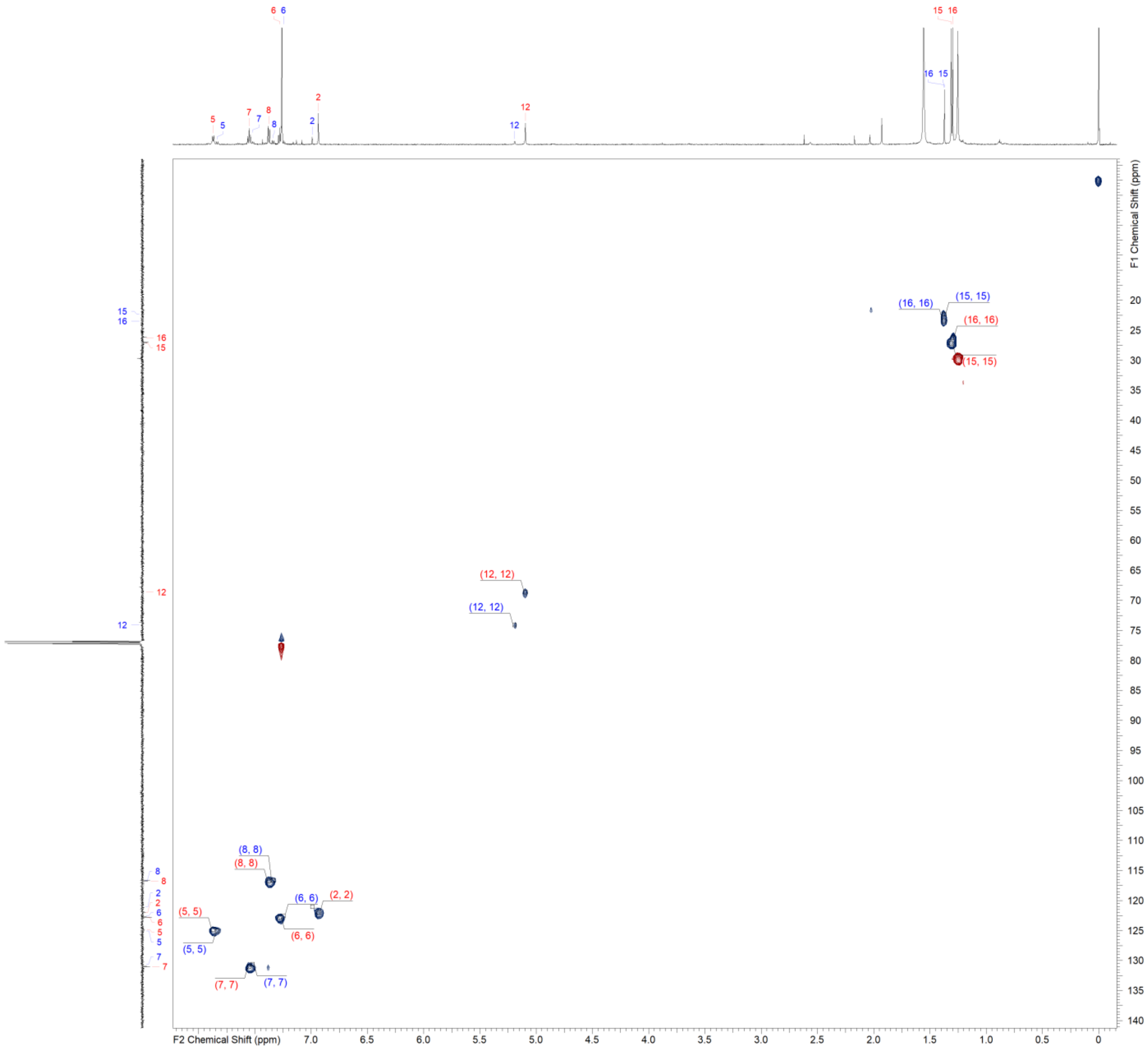

Figure S4. Fully assigned ^{1}H-^{13}C HSQC NMR spectrum of **6a,b** in $CDCl_3$ at 25 °C.

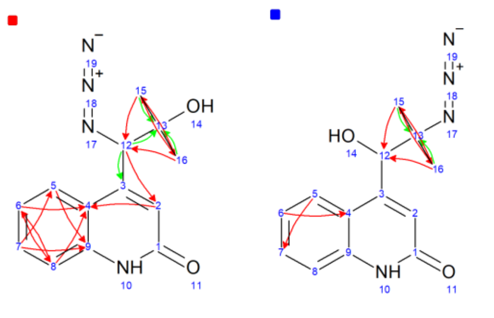

Acquisition Time (sec)	(0.1139, 0.0071)
Date	02 Apr 2022 15:08:44
Frequency (MHz)	(600.1300, 150.9028)
Nucleus	(1H, 13C)
Points Count	(2048, 1024)
Pulse Sequence	hmbcgplpndqf
Solvent	CHLOROFORM-d
Sweep Width (Hz)	(8988.42, 36185.27)
Constant (Hz)	10.0
Number of Transients	96
Spectrum Type	HMBC
Temperature (degree C)	25.000

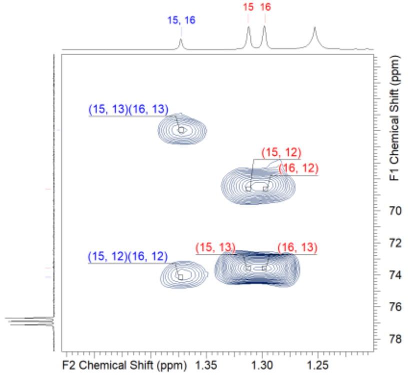

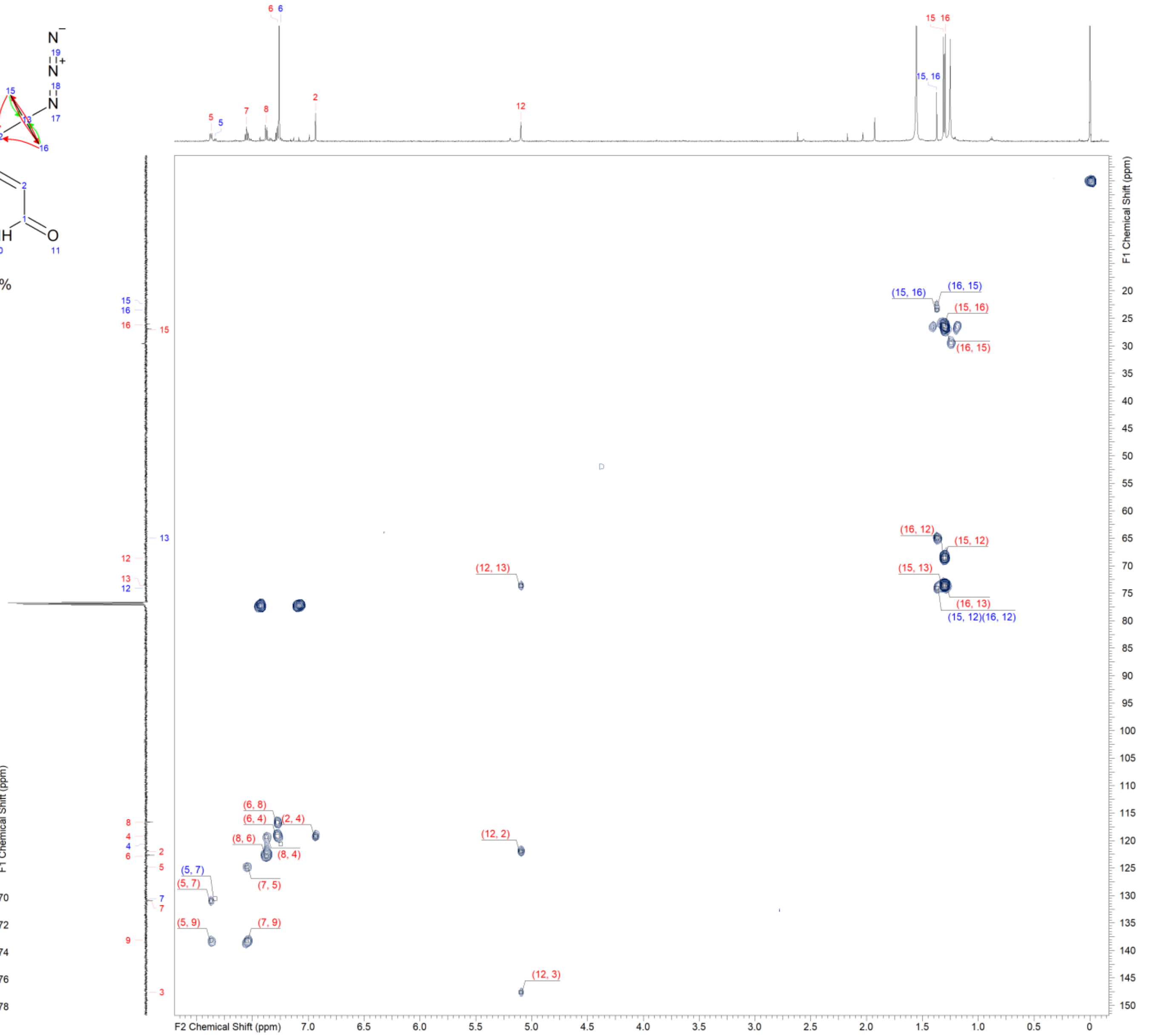

Figure S5. Fully assigned ^{1}H-^{13}C HMBC NMR spectrum of **6a,b** in $CDCl_3$ at 25 °C.

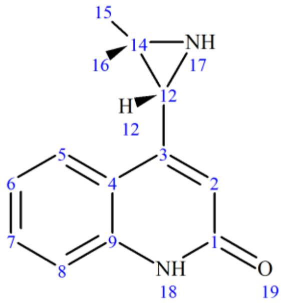

^{1}H NMR (600 MHz, CHLOROFORM-d): δ = 0.98 (s, 3 H, 15), 1.64 (s, 3 H, 16), 3.25 (br s, 1 H, 12), 6.81 (s, 1 H, 2), 7.25 (t, J = 7.0 Hz, 1 H, 6), 7.46 - 7.51 (m, 1 H, 8), 7.51 - 7.56 (m, 1 H, 7), 7.70 (br d, J = 7.7 Hz, 1 H, 5), 12.14 (br s, 1 H, 10) ppm

Shift1 (ppm)	H's	Type	J (Hz)	Atom1	Multiplet1
0.98	3.000	s	-	15	M01
1.64	3.000	s	-	16	M10
3.25	1.000	br s	-	12	M03
6.81	1.000	s	-	2	M04
7.25	1.000	t	7.0	6	M05
7.49	1.000	m	-	8	M06
7.52	1.000	m	-	7	M09
7.70	1.000	br d	7.7	5	M07
12.14	1.000	br s	-	10	M08

Solvent	CHLOROFORM-d
Pulse Sequence	zg30
Temperature (degree C)	25.000
Acquisition Time (sec)	1.3631
Number of Transients	64
Points Count	32768
Sweep Width (Hz)	12018.86
Spectrum Offset (Hz)	5393.7549
Original Points Count	16384
Nucleus	1H
Frequency (MHz)	600.1300

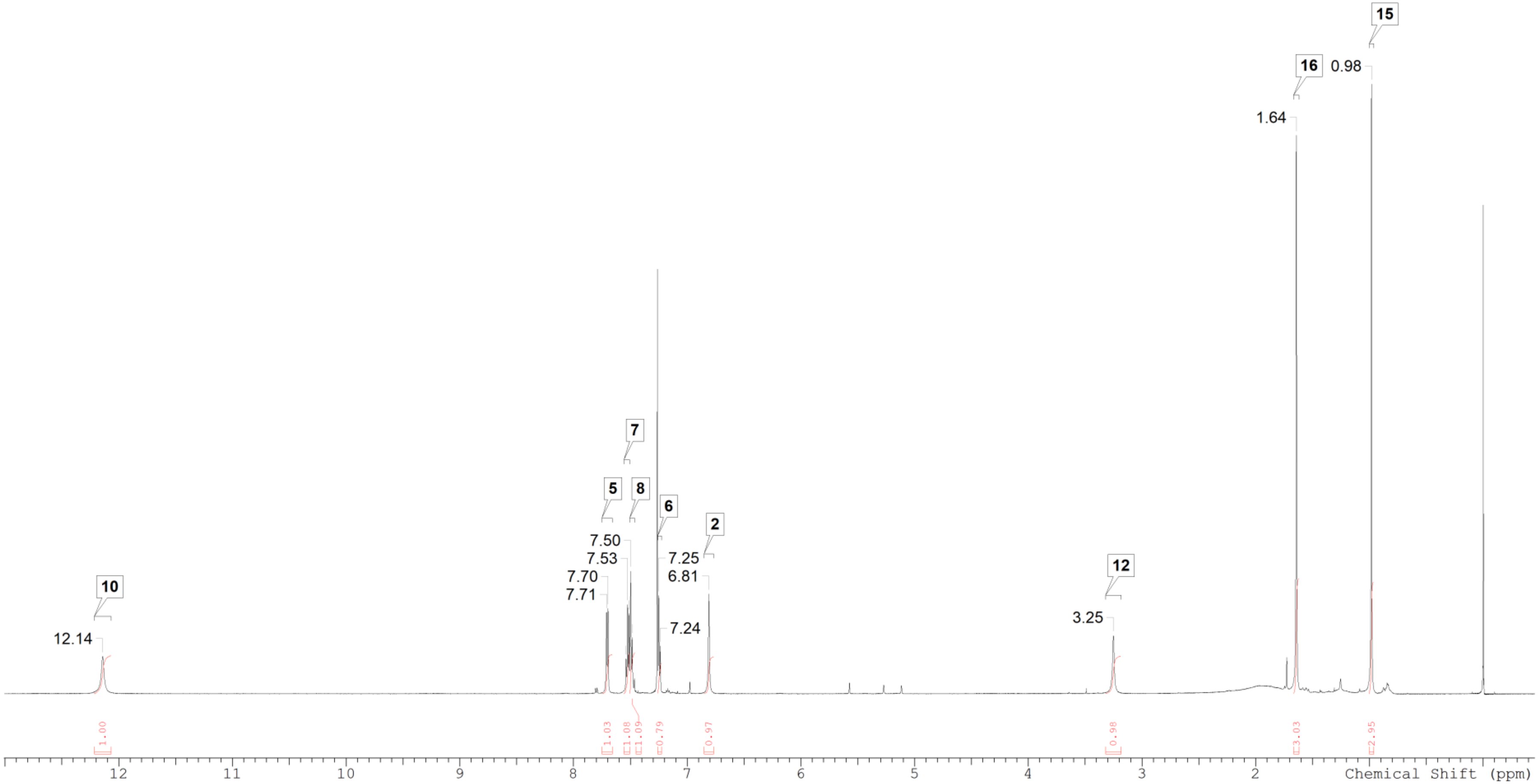

Figure S6. Structure, numbering and full assignment of ^{1}H NMR spectrum for compound **7** in $CDCl_3$ at 25 °C.

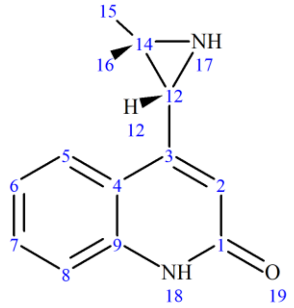

^{13}C NMR (151 MHz, CHLOROFORM-d): δ = 19.2 (15), 26.8 (16), 39.7 (14), 43.0 (12), 116.7 (8), 119.6 (2), 119.8 (4), 122.7 (6), 123.8 (5), 130.6 (7), 138.3 (9), 149.0 (3), 164.2 (1) ppm

Atom	Exp. Shift (ppm)
15	19.2
16	26.8
14	39.7
12	43.0
8	116.7
2	119.6
4	119.8
6	122.7
5	123.8
7	130.6
9	138.3
3	149.0
1	164.2

Solvent	**CHLOROFORM-d**
Pulse Sequence	**zgpg30**
Temperature (degree C)	**25.200**
Acquisition Time (sec)	**0.9110**
Number of Transients	**3072**
Points Count	**32768**
Sweep Width (Hz)	**35970.13**
Spectrum Offset (Hz)	**15091.0752**
Original Points Count	**32768**
Nucleus	**13C**
Frequency (MHz)	**150.9028**

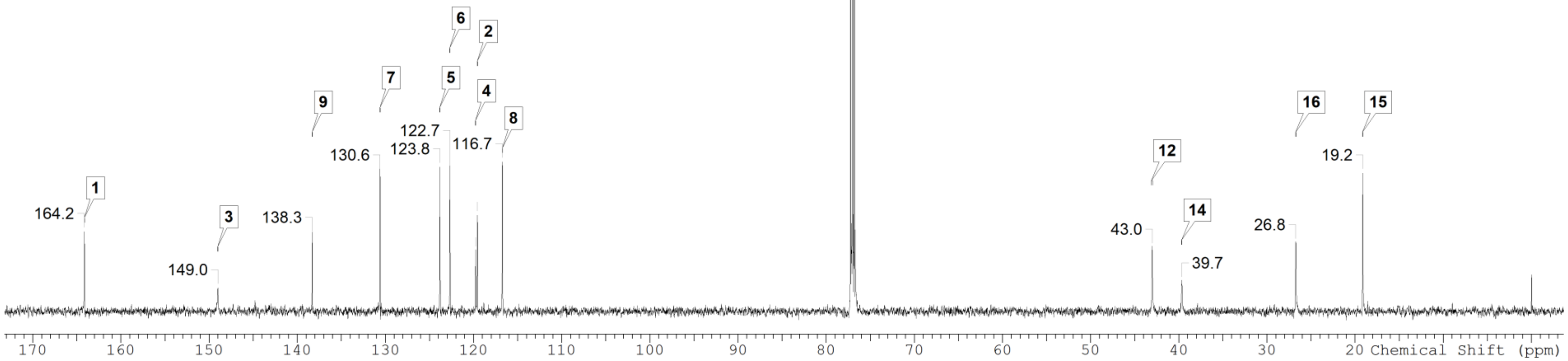

Figure S7. Structure, numbering and full assignment of ^{13}C NMR spectrum for compound **7** in $CDCl_3$ at 25 °C.

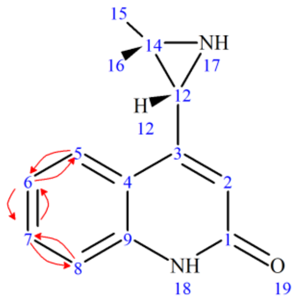

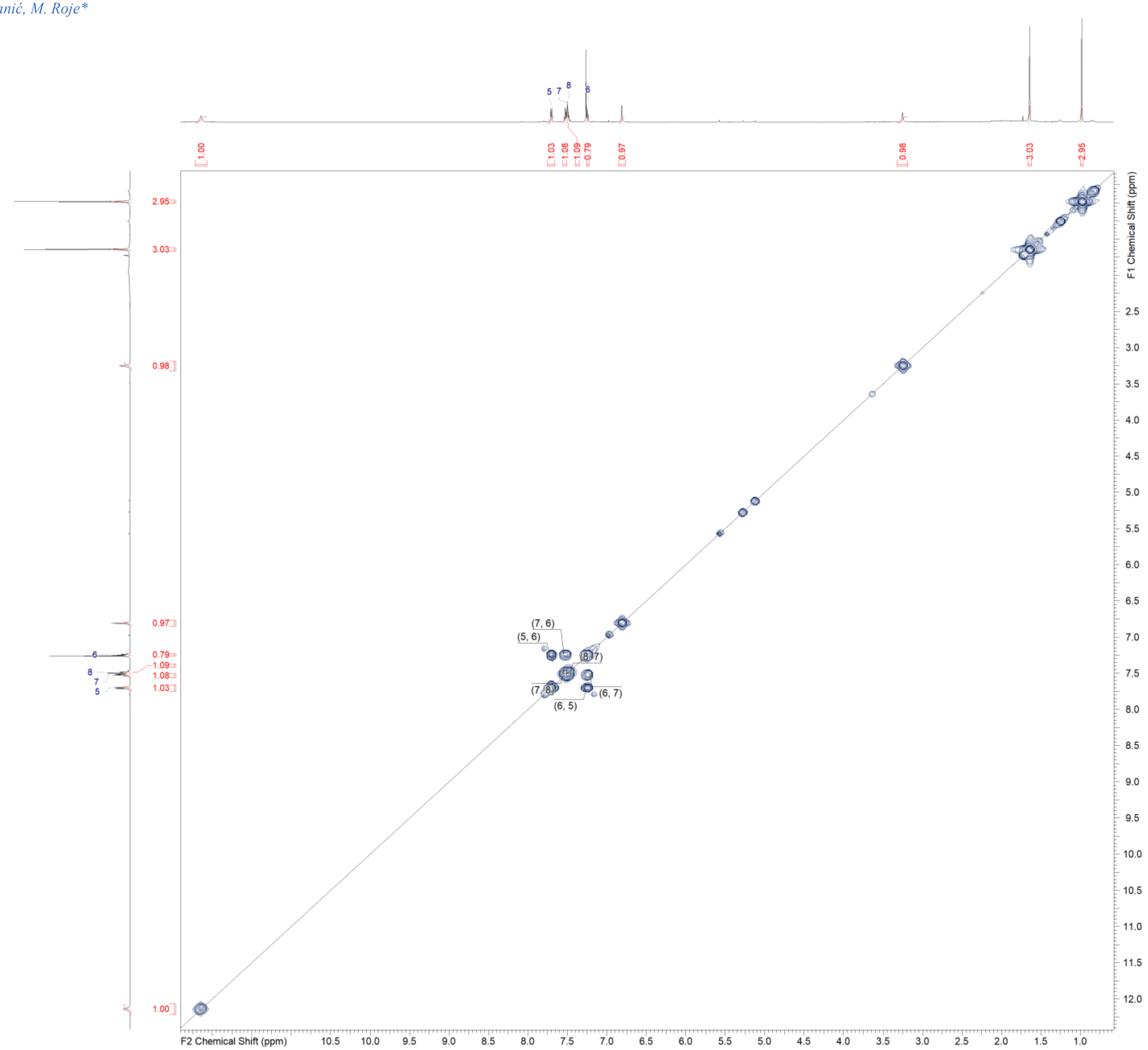

Acquisition Time (sec)	(0.1065, 0.0267)
Date	17 Mar 2022 17:00:12
Frequency (MHz)	(600.1300, 600.1300)
Nucleus	(1H, 1H)
Points Count	(2048, 2048)
Pulse Sequence	cosygpqf
Solvent	CHLOROFORM-d
Sweep Width (Hz)	(9610.69, 9597.45)
Number of Transients	2
Spectrum Type	COSY
Temperature (degree C)	25.000

Figure S8. Fully assigned ^{1}H-^{1}H COSY NMR spectrum of compound **7** in $CDCl_3$ at 25 °C.

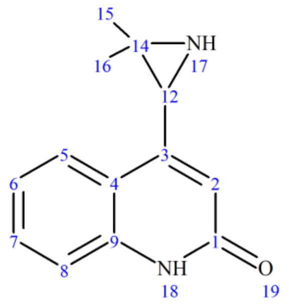

Acquisition Time (sec)	(0.1139, 0.0056)
Date	17 Mar 2022 17:00:14
Frequency (MHz)	(600.1300, 150.9028)
Nucleus	(1H, 13C)
Points Count	(1024, 1024)
Pulse Sequence	hsqcedetgpsisp.2
Solvent	CHLOROFORM-d
Sweep Width (Hz)	(8984.02, 27138.29)
Number of Transients	16
Spectrum Type	HSQC-DEPT
Temperature (degree C)	25.000

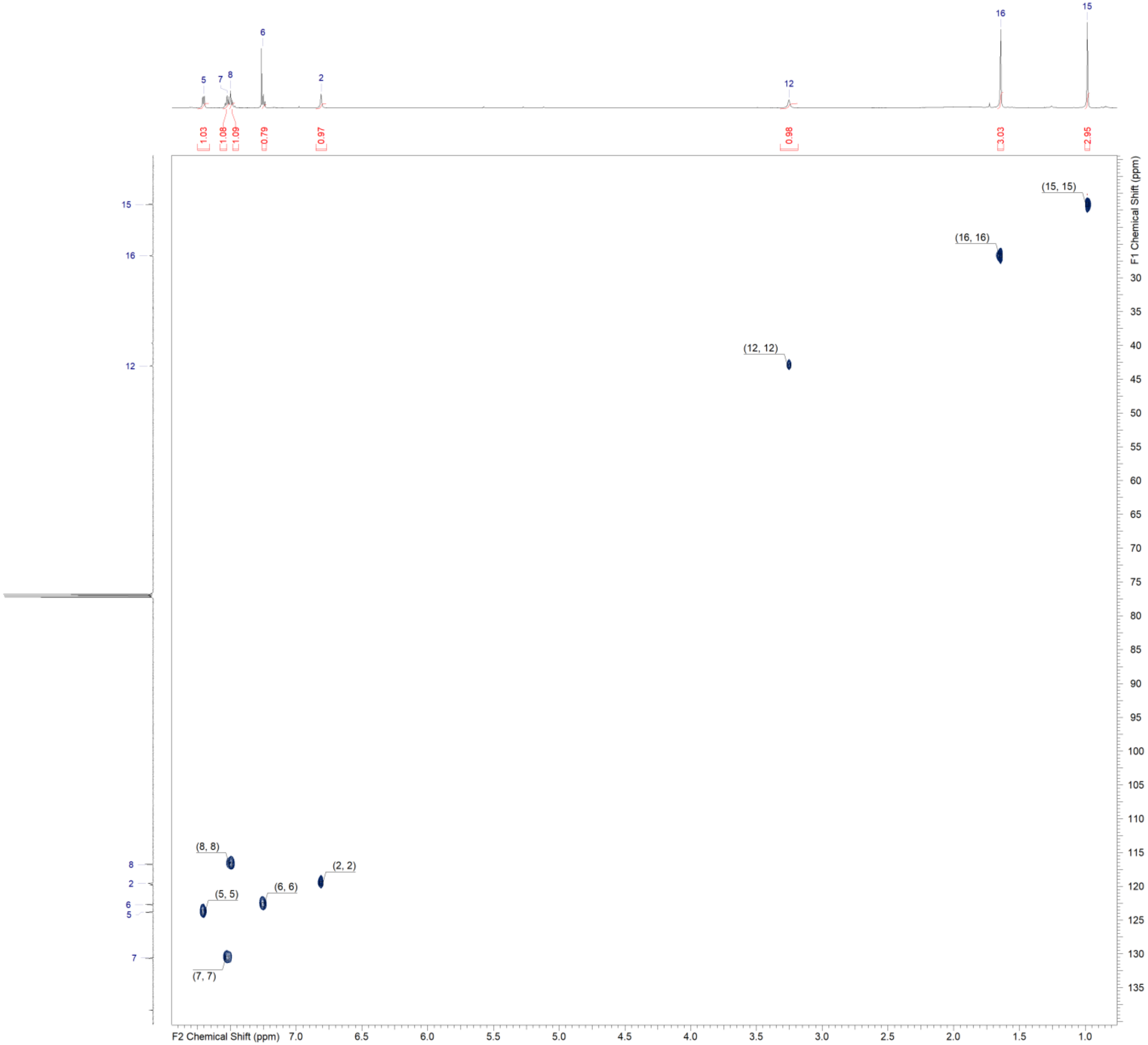

Figure S9. Fully assigned ^{1}H-^{13}C HSQC NMR spectrum of compound **7** in $CDCl_3$ at 25 °C.

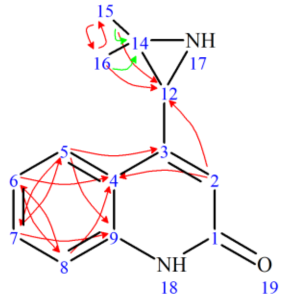

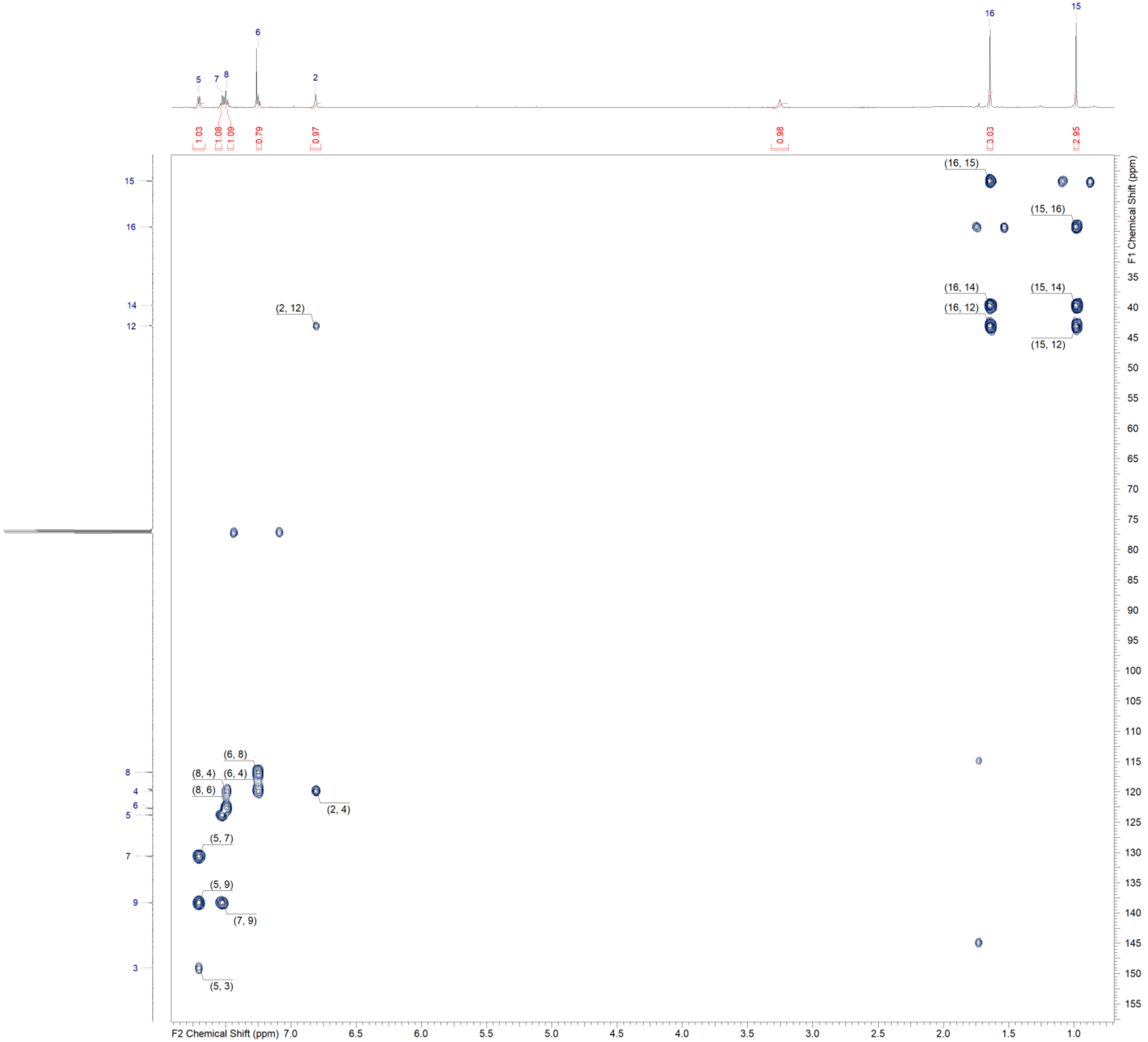

Acquisition Time (sec)	(0.1139, 0.0071)
Date	17 Mar 2022 17:00:18
Frequency (MHz)	(600.1300, 150.9028)
Nucleus	(1H, 13C)
Points Count	(2048, 1024)
Pulse Sequence	hmbcgplpndqf
Solvent	CHLOROFORM-d
Sweep Width (Hz)	(8988.42, 36185.27)
Constant (Hz)	10.0
Number of Transients	16
Spectrum Type	HMBC
Temperature (degree C)	25.000

Figure S10. Fully assigned ^{1}H-^{13}C HMBC NMR spectrum of compound **7** in $CDCl_3$ at 25 °C.

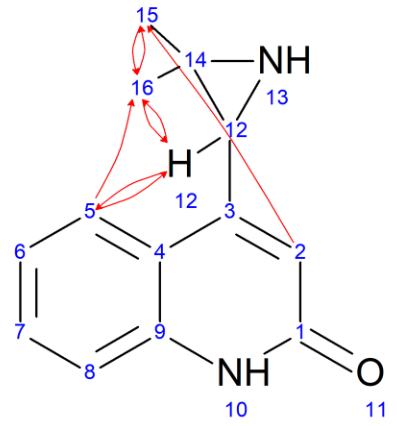

Acquisition Time (sec)	(0.1221, 0.0305)
Comment	marinoaziridin B CDCl3
Frequency (MHz)	(600.1300, 600.1300)
Nucleus	(1H, 1H)
Origin	spect
Original Points Count	(1024, 256)
Points Count	(2048, 1024)
Pulse Sequence	noesygpphzs
Solvent	CHLOROFORM-d
Sweep Width (Hz)	(8385.17, 8393.67)

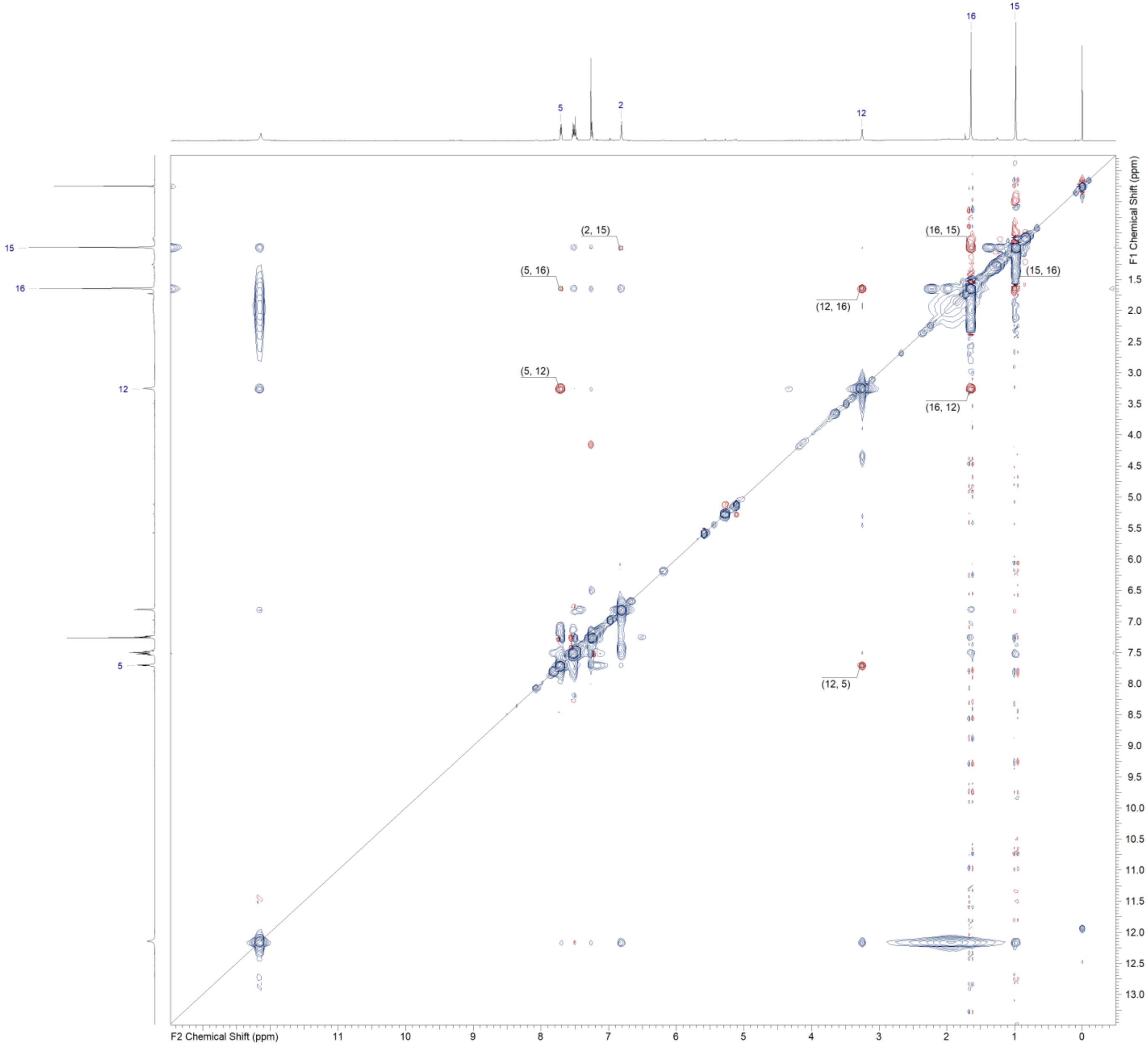

Figure S11. Fully assigned ^{1}H-^{1}H NOESY NMR spectrum of compound **7** in $CDCl_3$ at 25 °C.

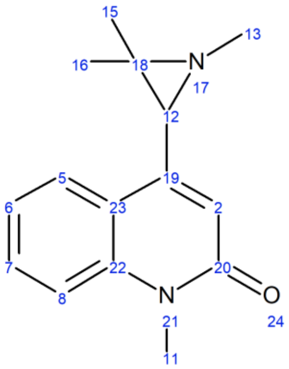

^{1}H NMR (600 MHz, CHLOROFORM-d): δ = 0.90 (s, 3 H, 16), 1.51 (s, 3 H, 15), 2.44 (s, 1 H, 12), 2.60 (s, 3 H, 13), 3.72 (s, 3 H, 11), 6.85 (d, J = 1.1 Hz, 1 H, 2), 7.25 (ddd, J = 8.1, 7.0, 1.2 Hz, 1 H, 6), 7.39 (d, J = 8.4 Hz, 1 H, 8), 7.58 (ddd, J = 8.5, 7.2, 1.5 Hz, 1 H, 7), 7.70 (dd, J = 7.9, 1.3 Hz, 1 H, 5) ppm

Shift1 (ppm)	H's	Type	J (Hz)	Atom1	Multiplet1
0.90	3.000	s	-	16	M09
1.51	3.000	s	-	15	M08
2.44	1.000	s	-	12	M10
2.60	3.000	s	-	13	M07
3.72	3.000	s	-	11	M06
6.85	1.000	d	1.1	2	M05
7.25	1.000	ddd	8.1, 7.0, 1.2	6	M04
7.39	1.000	d	8.4	8	M03
7.58	1.000	ddd	8.5, 7.2, 1.5	7	M02
7.70	1.000	dd	7.9, 1.3	5	M01

Solvent	**CHLOROFORM-d**
Pulse Sequence	**zg30**
Temperature (degree C)	**25.000**
Acquisition Time (sec)	**1.3631**
Number of Transients	**128**
Points Count	**32768**
Sweep Width (Hz)	**12018.86**
Spectrum Offset (Hz)	**5391.5166**
Original Points Count	**16384**
Nucleus	**1H**
Frequency (MHz)	**600.1300**

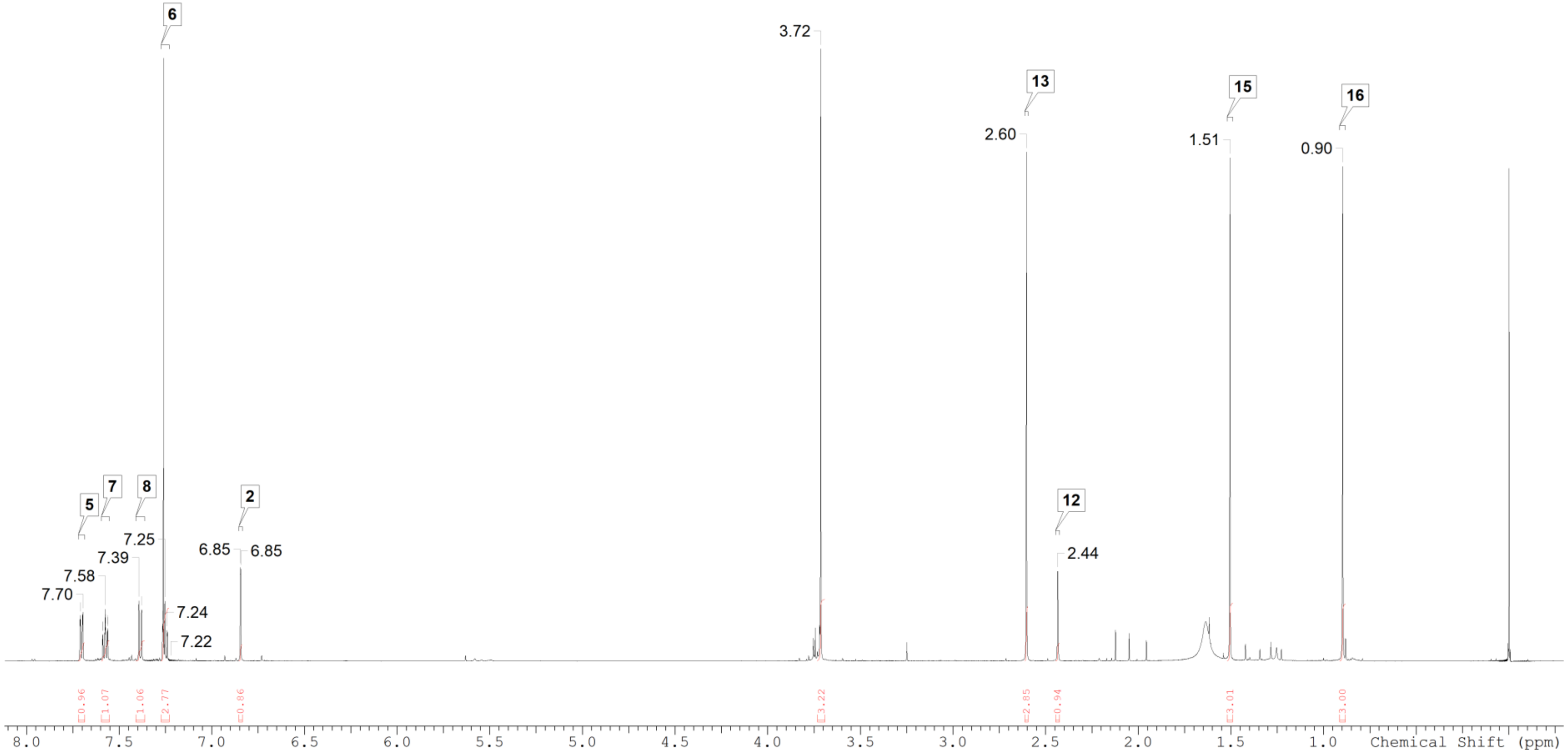

Figure S12. Structure, numbering and full assignment of ^{1}H NMR spectrum for compound **8** in $CDCl_3$ at 25 °C.

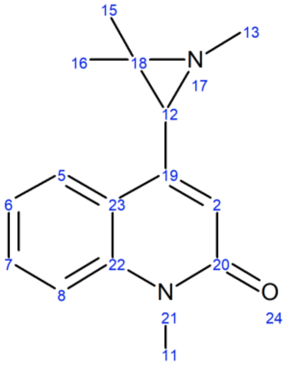

^{13}C NMR (151 MHz, CHLOROFORM-d): δ = 17.3 (15), 21.4 (16), 29.3 (11), 39.5 (13), 43.1 (14), 51.3 (12), 114.5 (8), 119.9 (2), 120.8 (4), 121.9 (6), 124.6 (5), 130.3 (7), 139.9 (9), 146.9 (3), 162.3 (1) ppm

Shift1 (ppm)	C's	Type	Atom1	Multiplet1
17.34	1.000	s	15	M01
21.35	1.000	s	16	M02
29.25	1.000	s	11	M03
39.47	1.000	s	13	M04
43.09	1.000	s	14	M05
51.27	1.000	s	12	M06
114.53	1.000	s	8	M07
119.91	1.000	s	2	M08
120.76	1.000	s	4	M09
121.86	1.000	s	6	M10
124.56	1.000	s	5	M11
130.34	1.000	s	7	M12
139.88	1.000	s	9	M13
146.93	1.000	s	3	M14
162.33	1.000	s	1	M15

Solvent	**CHLOROFORM-d**
Pulse Sequence	**deptqgpsp.2**
Temperature (degree C)	**25.100**
Acquisition Time (sec)	**0.5999**
Number of Transients	**8192**
Points Count	**32768**
Sweep Width (Hz)	**39368.88**
Spectrum Offset (Hz)	**15089.8027**
Original Points Count	**23619**
Nucleus	**13C**
Frequency (MHz)	**150.9028**

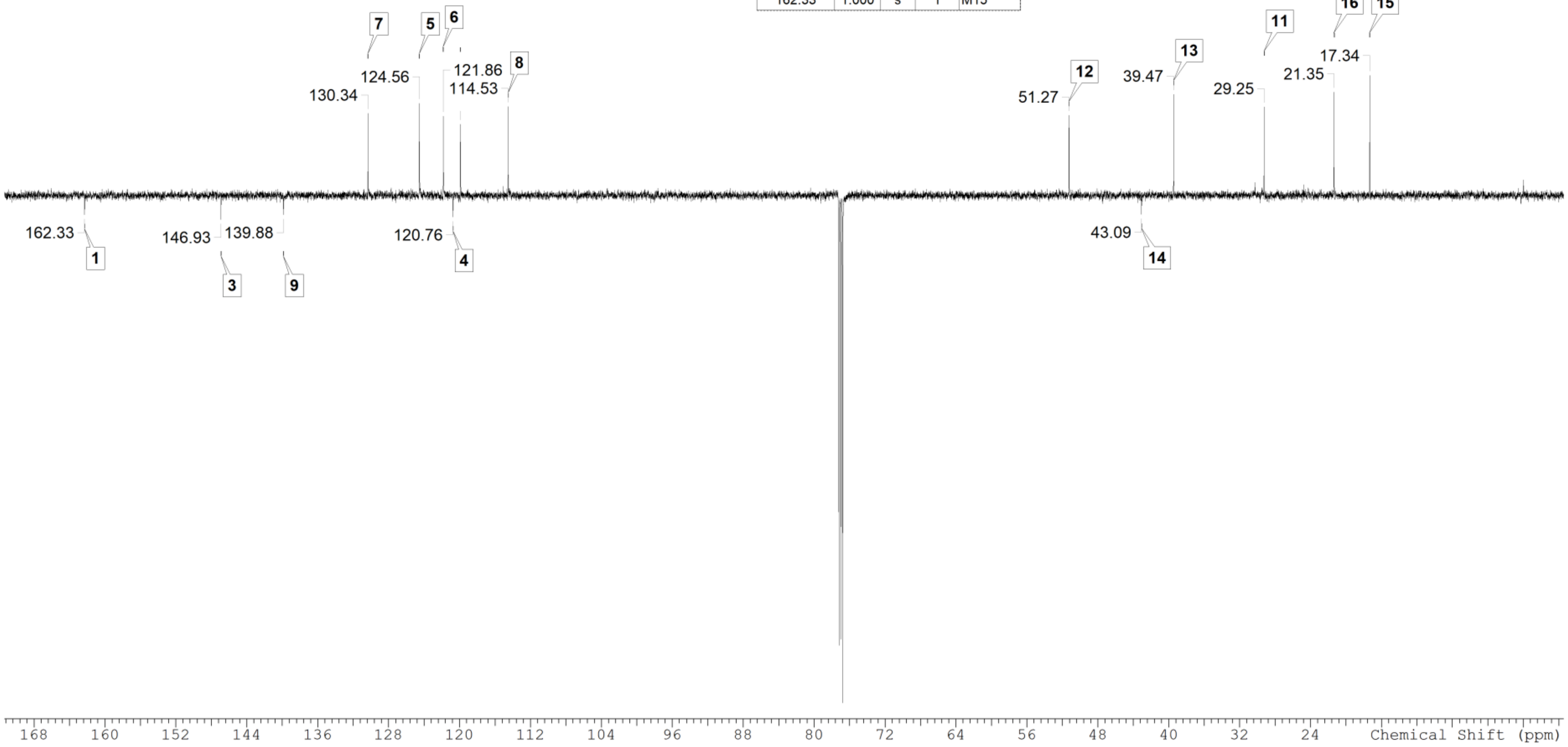

Figure S13. Structure, numbering and full assignment of ^{13}C NMR spectrum for compound **8** in $CDCl_3$ at 25 °C.

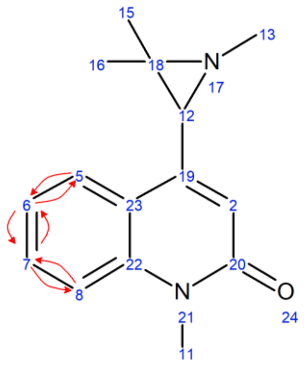

Acquisition Time (sec)	(0.1065, 0.0267)
Date	29 Aug 2022 18:52:16
Frequency (MHz)	(600.1300, 600.1300)
Nucleus	(1H, 1H)
Points Count	(2048, 2048)
Pulse Sequence	cosygpqf
Solvent	CHLOROFORM-d
Sweep Width (Hz)	(9610.69, 9597.45)
Number of Transients	4
Spectrum Type	COSY
Temperature (degree C)	25.000

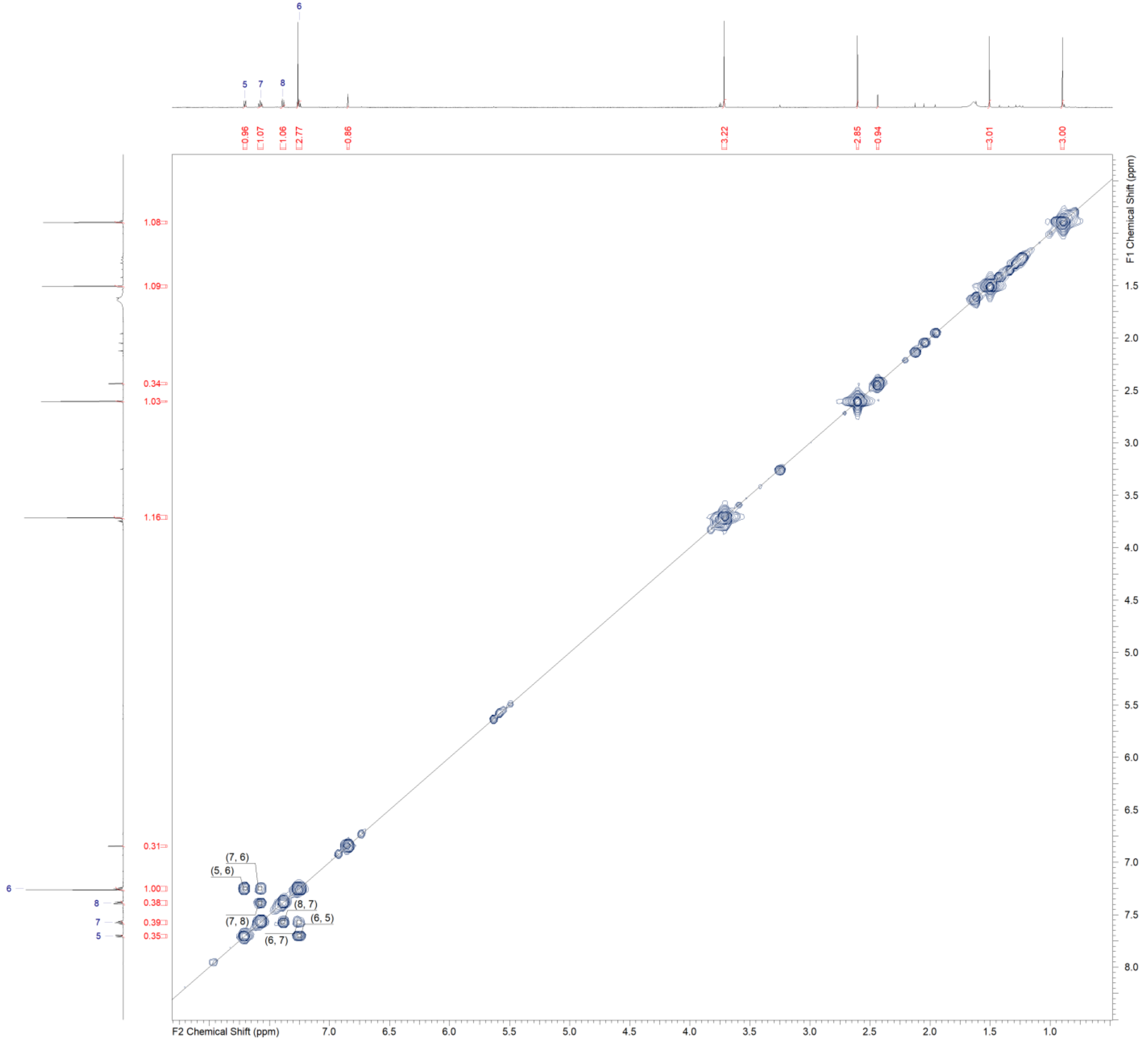

Figure S14. Fully assigned ^{1}H-^{1}H COSY NMR spectrum of compound **8** in $CDCl_3$ at 25 °C.

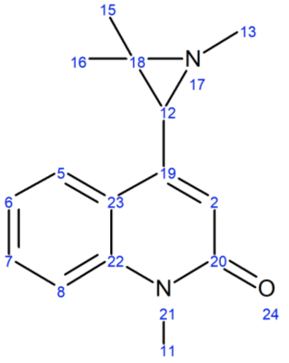

Acquisition Time (sec)	(0.1139, 0.0094)
Date	30 Aug 2022 21:05:14
Frequency (MHz)	(600.1300, 150.9028)
Nucleus	(1H, 13C)
Points Count	(1024, 1024)
Pulse Sequence	hsqcedetgpsisp.2
Solvent	CHLOROFORM-d
Sweep Width (Hz)	(8984.02, 27138.29)
Number of Transients	64
Spectrum Type	HSQC-DEPT
Temperature (degree C)	25.100

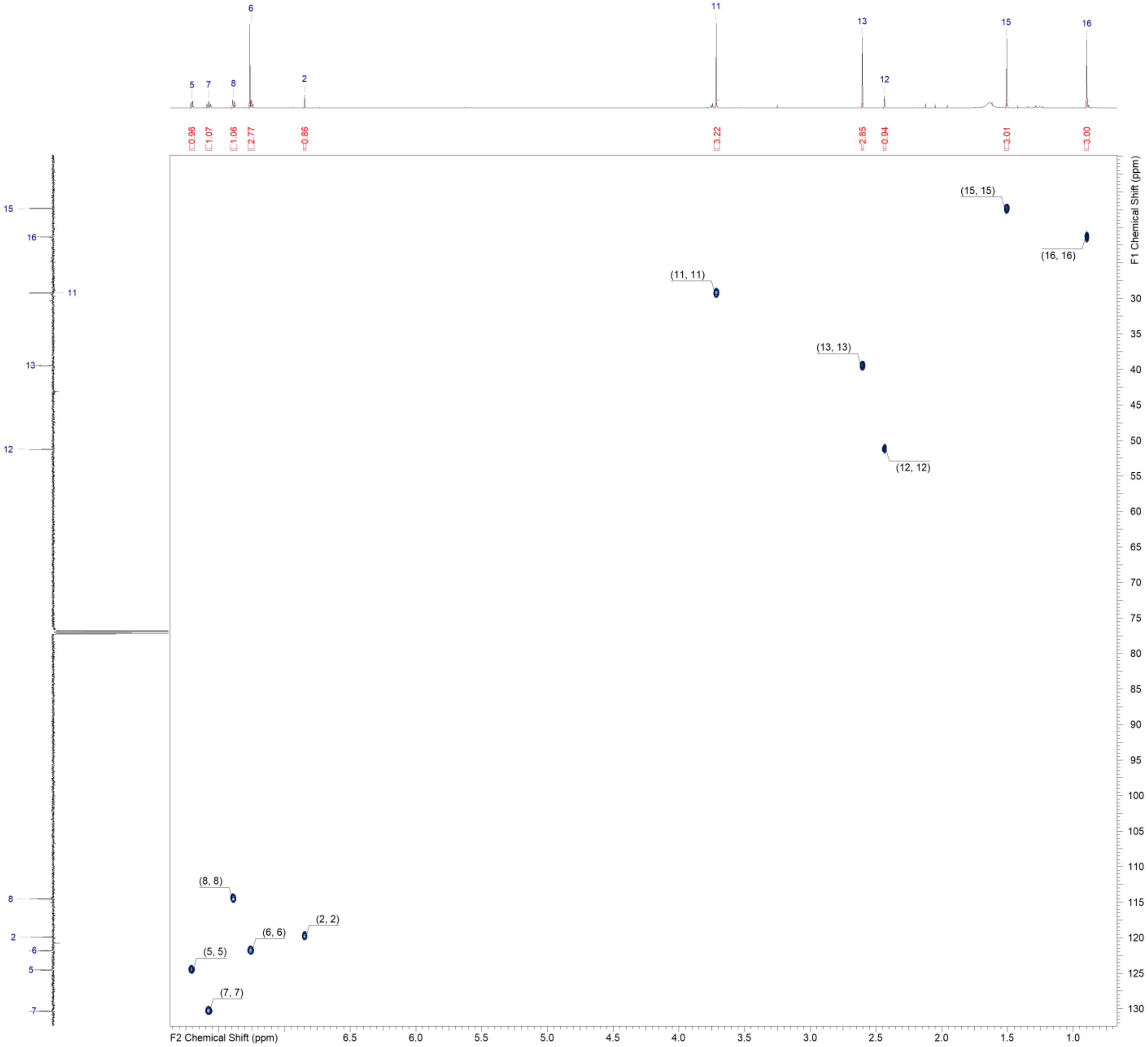

Figure S15. Fully assigned ^{1}H-^{13}C HSQC NMR spectrum of compound **8** in $CDCl_3$ at 25 °C.

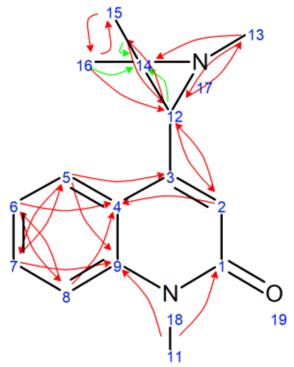

Acquisition Time (sec)	(0.1139, 0.0071)
Date	29 Aug 2022 18:52:22
Frequency (MHz)	(600.1300, 150.9028)
Nucleus	(1H, 13C)
Points Count	(2048, 1024)
Pulse Sequence	hmbcgplpndqf
Solvent	CHLOROFORM-d
Sweep Width (Hz)	(8988.42, 36185.27)
Constant (Hz)	10.0
Number of Transients	96
Spectrum Type	HMBC
Temperature (degree C)	24.900

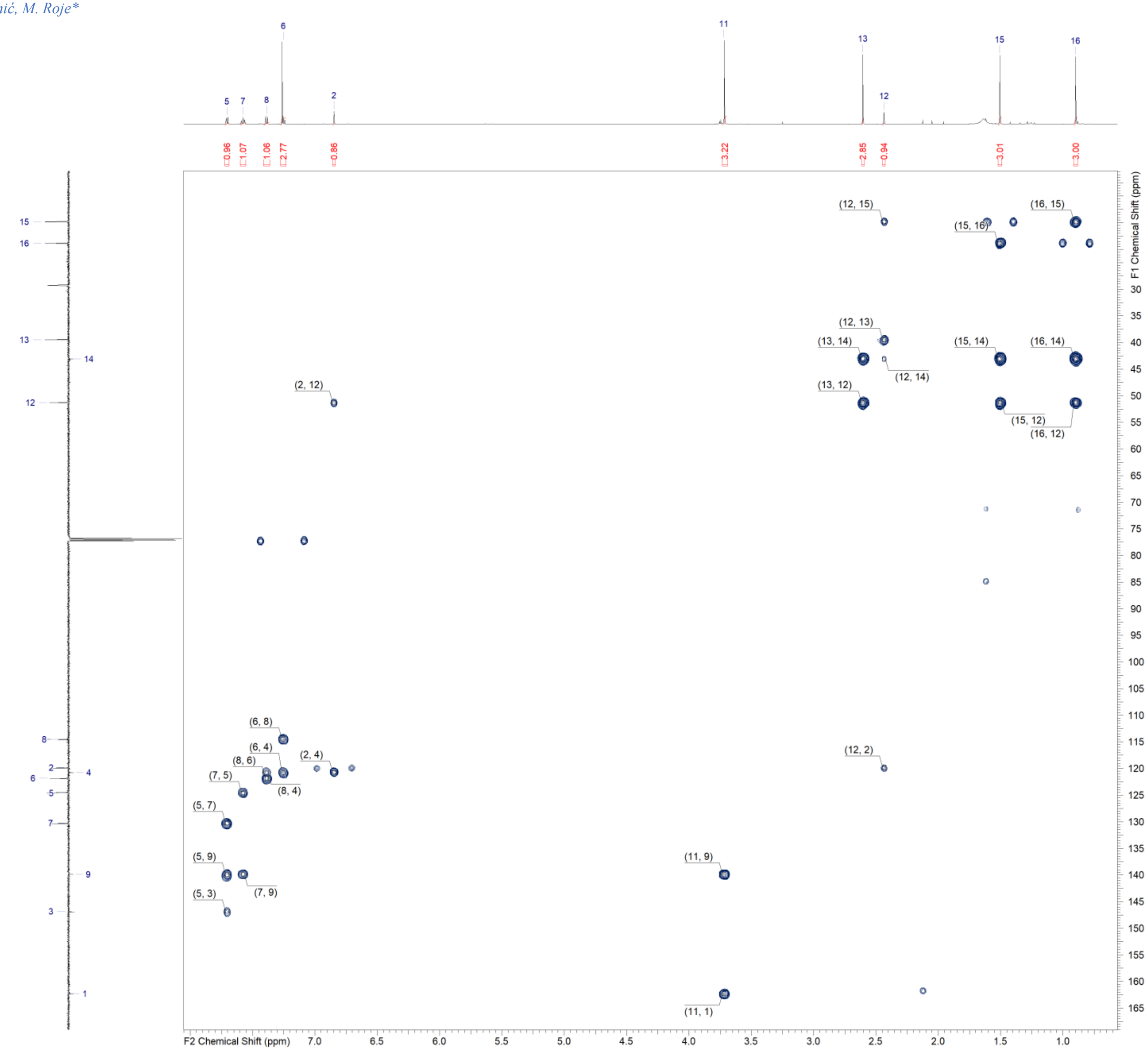

Figure S16. Fully assigned ^{1}H-^{13}C HMBC NMR spectrum of compound **8** in $CDCl_3$ at 25 °C.

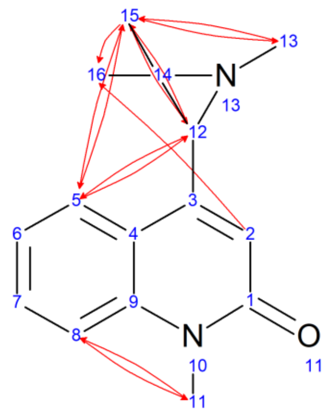

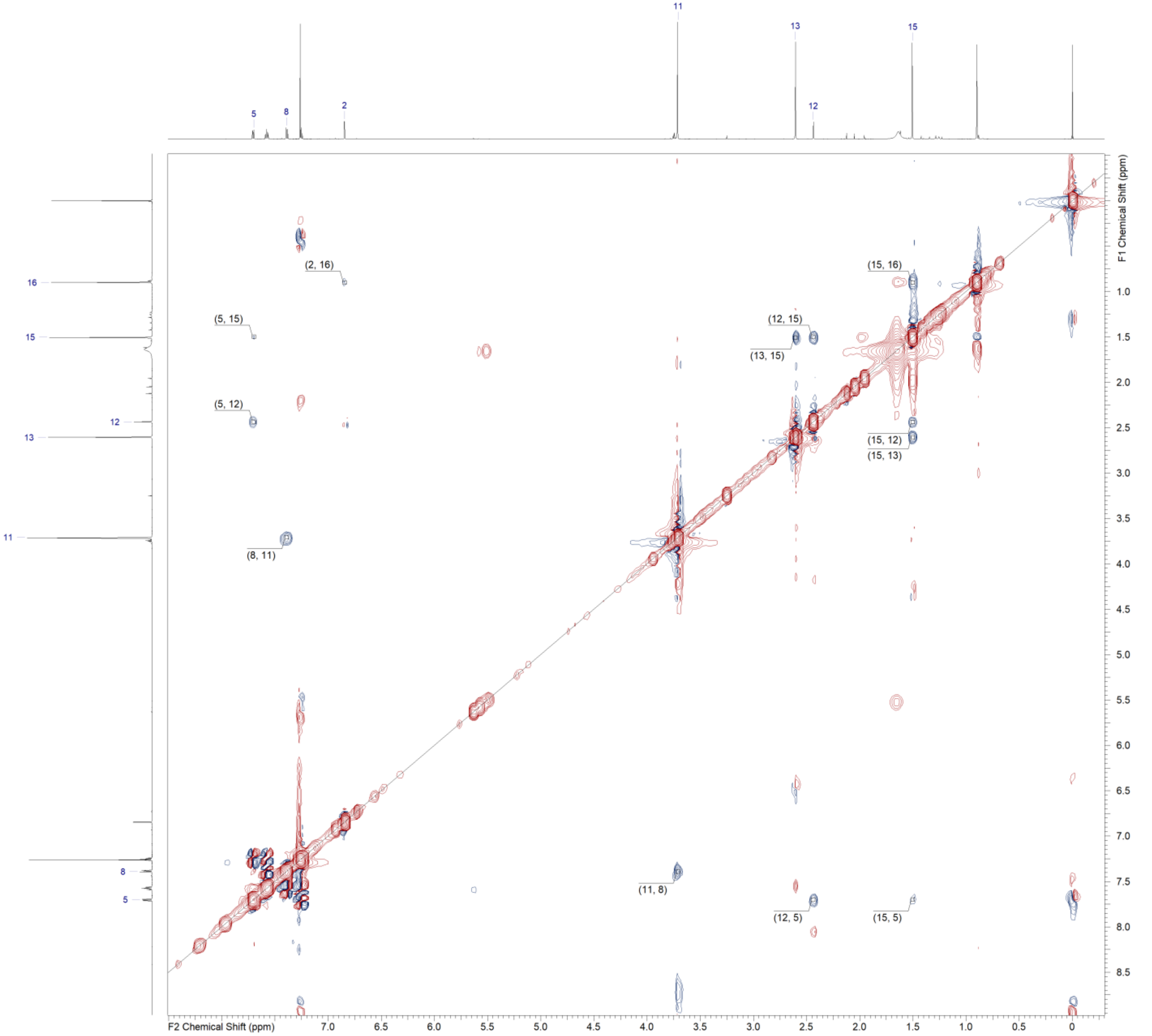

Acquisition Time (sec)	(0.1659, 0.0830)
Comment	marinoaziridine a
Frequency (MHz)	(300.1300, 300.1300)
Nucleus	(1H, 1H)
Origin	spect
Original Points Count	(1024, 512)
Points Count	(2048, 2048)
Pulse Sequence	noesyph
Solvent	CHLOROFORM-d
Sweep Width (Hz)	(6169.83, 6167.72)

Figure S17. Fully assigned ^{1}H-^{1}H NOESY NMR spectrum of compound **8** in $CDCl_3$ at 25 °C.

3.3. Comparison of synthetic and isolated marinoaziridne B with literature data

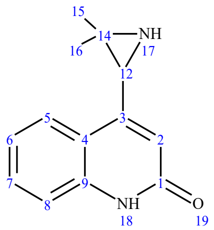

marinoaziridine B (7)

Table S1. Comparison of ^{1}H NMR chemical shifts[a] of synthetic structure and literature data (Fenical's isolation report) [2]

position	Fenical's isolation report (ppm)[2]	Synthetic structure (ppm)	$\Delta\delta^b$
**15**	0.95	0.98	0.03
**16**	1.43	1.64	0.21
**12**	4.27	3.25	-1.02
**2**	6,10	6.81	0.71
**6**	6.75	7.25	0.50
**8**	6.59	7.49	0.90
**7**	7.17	7.52	0.35
**5**	7.45	7.71	0.26

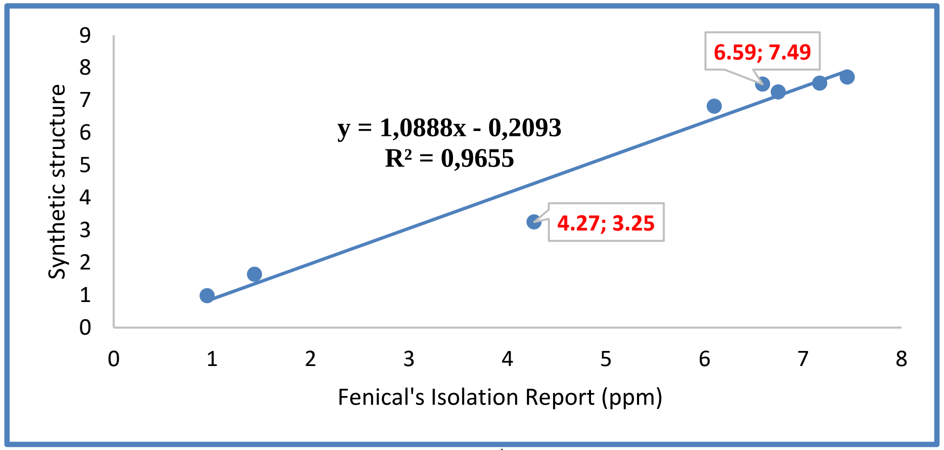

[a]NMR spectra were recorded in $CDCl_3$ at 25°C, [b]$\Delta\delta_1 = \delta_{(synthetic\ structure)} - \delta_{(Fenical's\ Isolation\ Repor}$

Figure S18. Linear correlations of ^{1}H NMR chemical shifts ($CDCl_3$) for Fenical's isolation report and synthetic structure.

Table S2. Comparison of ^{13}C NMR data[a] of synthetic structure and literature data (Fenical's Isolation Report) [2].

position	Fenical's isolation report (ppm) [2]**Error! Reference source not found.**	Proposed structure (synthetic) (ppm)	$\Delta\delta^{b}$
**15**	19.2	19.16	-0.04
**16**	29.7	26.75	-2.95
**14**	56.2	39.67	-16.53
**12**	63.5	43.03	-20.47
**8**	115.5	116.74	1.24
**2**	113.7	119.58	5.88
**4**	114.1	119.79	5.69
**6**	119.3	122.70	3.40
**5**	126.8	123.83	-2.97
**7**	132.0	130.63	-1.37
**9**	144.1	138.32	-5.78
**3**	156.3	149.03	-7.27
**1**	177.0	164.16	-12.84

[a]NMR spectra were recorded and calculated in $CDCl_3$ at 25°C; [b]$\Delta\delta_1 = \delta_{\text{(proposed structure)}} - \delta_{\text{(Fenical's Isolation Report)}}$.

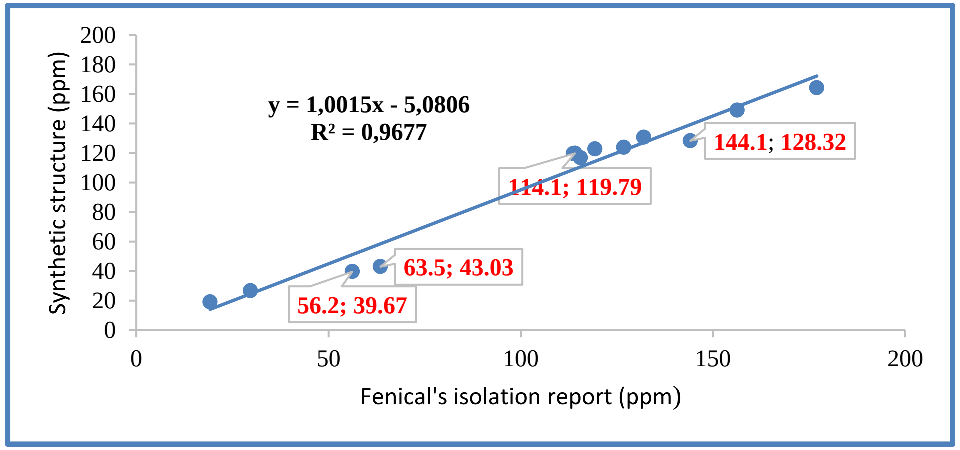

Figure S19. Linear correlations of ^{13}C NMR chemical shifts ($CDCl_3$) for: a) Fenical's isolation report and synthetic structure.

4. Biological Activity

a. *In silico* ADMET and bioactivity profiling

Table S3. Physicochemical parameters all predicted by ADMET Predictor ver. 10.0.0.11.

Identifier[1]	Basic_pKa[2]	FCation[3]	logP[4]	logD[5]	Peff[6]	MDCK[7]	Sw[8]	BBB_Filter[9]	LogBB[10]	hum_fup%[11]	rat_fup%[12]	Vd[13]	RBP[14]	RBP_rat[15]	Fumic[16]
rac-**7**	8.240	0.873	1.625	0.736	2.009	372.070	2.192	High (99%)	0.078	67.635	40.166	2.720	1.173	1.427	0.777
rac-**8**	8.430	0.916	1.855	0.785	3.662	933.479	4.071	High (99%)	0.453	68.371	44.718	2.449	1.092	1.473	0.749

[1]Compound identifier. [2]Predicted macroscopic pKa that appear to be dominated by basic functional groups. [3]Cumulative contribution of purely cationic species to fraction ionized at pH defined on input (pH =7.4). [4]Admet predictor model for log P with RMSE/MAE = 0.30/0.23. [5]log D at 7.4 RMSE/MAE = 0.56/0.41. [6]Effective human jejunal permeability (cm/s x 10^4). RMSE/MAE = 0.31/0.25 log units. [7]Apparent MDCK Transwell permeability (cm/s x 10^7). RMSE/MAE = 0.47/0.48 (2D and 3D) log units. [8]Water solubility (mg/mL). RMSE/MAE = 0.59/0.45 (2D) and 0.57/0.43 (3D) in log units. [9]Predicts whether or not a compound can penetrate the Blood Brain Barrier. Overall accuracy = 92%. [10]Logarithm of the Brain/Blood partition coefficient. RMSE/MAE = 0.37/0.28 (2D and 3D). [11]Percent UNBOUND to blood plasma proteins in human. RMSE/MAE = 0.43/0.32 (2D) log units. [12]Percent UNBOUND to blood plasma proteins in Rat. RMSE/MAE = 0.30/0.24 log units. [13]Volume of distribution (L/kg) in human at steady state. RMSE/MAE = 0.40/0.30 log units (2D and 3D). [14]Blood to plasma concentration ratio in human. RMSE/MAE = 0.09/0.07 (2D) log units. [15]Blood to plasma concentration ratio in rat. RMSE/MAE = 0.10/0.08 (2D) log units. [16]Fraction unbound in human liver microsomes at 1 mg/mL microsomal protein concentration. RMSE/MAE = 0.18/0.13.

Table S4. Toxicity parameters predicted by ADMET Predictor ver. 10.0.0.11.

Identifier[1]	Estro_Filter[2]	Andro_Filter[3]	Minnow_LC50[4]	Th_pyr_pIGC50[5]	Daphnia_LC506	Bioconcn[7]	Biodegradn[8]	hERG_Filter[9]	Rat_Acute[10]	Rat_TD50[11]	Mouse_TD50[12]	Chrom_Aberr[13]	PLipidosis[14]	Repro_Tox[15]	MUT_NIHS[16]
rac-**7**	Nontoxic (80%)	Nontoxic (57%)	38.965	0.209	1.026	2.634	No (95%)	No (64%)	332.625	18.919	205.33	Nontoxic (54%)	Nontoxic (69%)	Toxic (57%)	Positive (24%)
rac-**8**	Nontoxic (51%)	Toxic (53%)	13.609	0.653	4.899	6.351	No (95%)	Yes (99%)	361.796	29.323	99.433	Nontoxic (46%)	Toxic (69%)	Nontoxic (79%)	Positive (26%)

[1]Compound identifier. [2]Predicts whether or not the compound possesses estrogen receptor toxicity in rat. Overall accuracy = 90% (2D) and 95% (3D). [3]Predicts whether or not the compound possesses androgen receptor toxicity in rat. Overall accuracy = 82%. [4]LC_{50} for fathead minnow lethal toxicity (mg/L) after 96 hours of exposure. RMSE/MAE = 0.50/0.38 (2D and 3D) in molar log units. [5]Acute toxicity in Tetrahymena pyriformis protozoa expressed as -log of the 50% growth inhibitory concentration, (pIGC50) in mmol/L. RMSE/MAE = 0.32/0.25 (2D) and 0.32/0.24 (3D) in log units. [6]Acute toxicity in Daphnia magna (water fleas) expressed as the 50% lethality after 48 hours, (LC50) in mg/L. Overall RMSE/MAE = 0.79/0.62 (2D and 3D) in molar log units. [7]Bioconcentration factor - a partition coefficient between fish tissues and environmental water at steady state as concentration ratio (Cfish/Cwater). RMSE/MAE = 0.47/0.36 (2D and 3D) in log units. [8]Predicts whether or not the compound undergoes biodegradation readily in terms of relative biological oxygen demand (BOD>60%). Overall accuracy = 84%. [9] Predicts whether or not the compound blocks the hERG potassium channel. Overall accuracy = 88%. [10]LD_{50} for rat acute toxicity (mg/kg in oral dose that would be lethal to 50% of the rats). RMSE/MAE = 0.60/0.45 (2D) and 0.58/0.44 (3D) in log units. [11]TD_{50} for rat carcinogenicity (mg/kg/day in oral dose) over a standard lifetime. RMSE/MAE = 0.54/0.42 (2D) and 0.52/0.43 (3D) in log units. [12]TD_{50} for mouse carcinogenicity (mg/kg/day in oral dose) over a standard lifetime. RMSE/MAE = 0.47/0.38 (2D) and 0.46/0.38 (3D) in log units. [13]Predicts whether or not the compound will trigger the mutagenic chromosomal aberrations. Overall accuracy = 80%. [14]Qualitative estimation of causing phospholipidosis. Overall accuracy = 96%. [15]Qualitative estimation of reproductive / developmental toxicity. Overall accuracy = 90%. [16]Predicts the mutagenicity of the compound based on data compiled in 2017 by the National Institute of Health Sciences (NIHS) of Japan. Overall accuracy = 76%. Prediction confidence estimates are listed in parentheses.

b. Antiproliferative effect

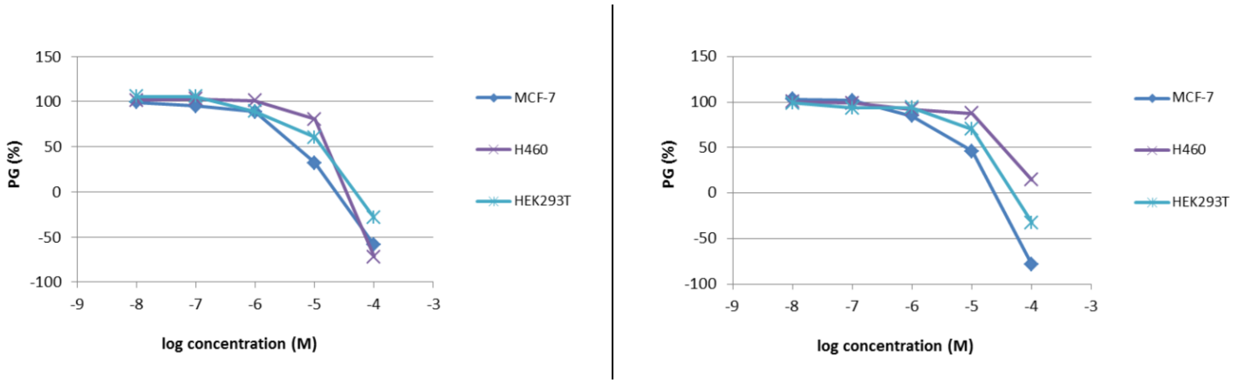

Figure S20. Dose-response profiles for compound *rac*-**7** and compound *rac*-**8** tested *in vitro* on H460, HEK293T and MCF-7.

Table S5. GI_{50} values (in µM).

	GI_{50}[a] (µM)		
**Compound ID**	**Cell lines**		
	**H460**	**HEK293T**	**MCF7**
compound **7**	16±0.2	13±0.1	5±1.0
compound **8**	33±1.0	15±4.0	7±5.0

[a]GI_{50}; the concentration that causes 50% growth inhibition.

c. Testing of embryotoxicity using the zebrafish model organism (*Danio rerio)*

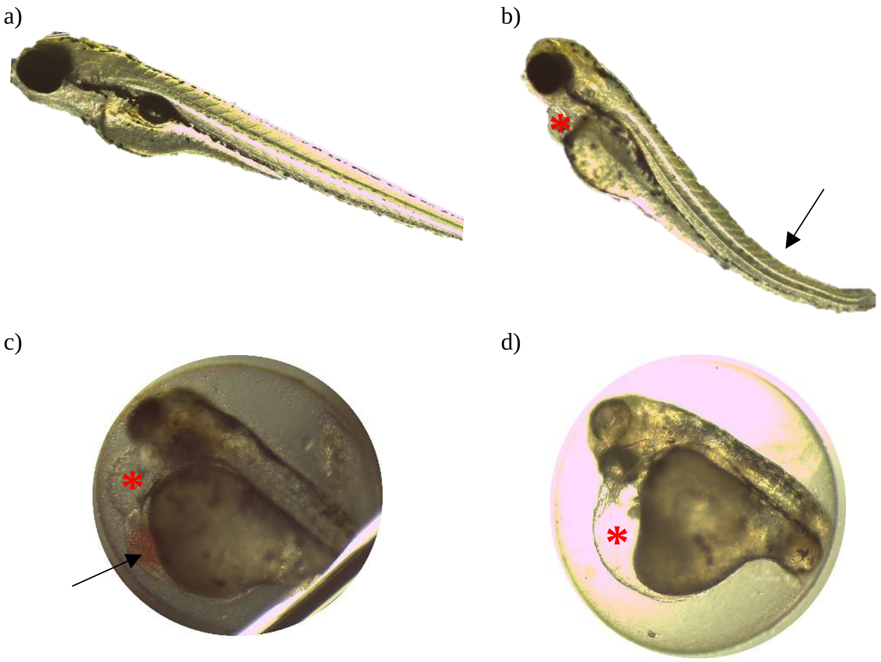

Figure S21. Developmental abnormalities in *Danio rerio* zebrafish exposed to the tested samples of compound *rac*-**7** and *compound rac*-**8** for 96 hours: a) normally developed larvae in the control groups, b) scoliosis (arrow), pericardial edema (star), c) bleeding in the yolk sac area (arrow), pericardial edema (star) d) edema in the area of the yolk sac (asterisk).

Table S6. Rate (%) of mortality, developmental abnormalities and hatching success of zebrafish *D. rerio* (*N* = 30) after 96 h of exposure to different concentrations of the tested compounds. Results are presented as mean value ± standard deviation (SD).

**Compound**		**Concentration**		
		**0.1mM**	**0.2 mM**	**0.4 mM**
rac-**7**	mortality rate (%)	100.00±0.00	100.00±0.00	100.00±0.00
	abnormality rate (%)	-	-	-
	hatching rate (%)	-	-	-
rac-**8**	mortality rate (%)	0.00±0.00	0.00±0.00	76.67±5.77
	abnormality rate (%)	0.00±0.00	6.67±11.55	100.00±0.00
	hatching rate (%)	100.00±0.00	23.33±5.77	0.00±0.00

5. References

1. Buljan A.**;** Roje M. Application of green chiral chromatography in enantioseparation of newly synthesized racemic marinoepoxides. *Mar. Drugs* **2022**, *20*, 530.
2. Choi E. J.; Nam S.-J.; Paul L.; Beatty D.; Kauffman C. A.; Jensen P. R.; Fenical W. Previously uncultured marine bacteria linked to novel alkaloid production. *Chem. and Biol.* **2015**, *22*, 1270–1279.
